# Effects of microglial depletion and TREM2 deficiency on Aβ plaque burden and neuritic plaque tau pathology in 5XFAD mice

**DOI:** 10.1186/s40478-021-01251-1

**Published:** 2021-09-09

**Authors:** Argyro Thalia Delizannis, Annelies Nonneman, Wangchen Tsering, An De Bondt, Ilse Van den Wyngaert, Bin Zhang, Emily Meymand, Modupe F. Olufemi, Pyry Koivula, Shaniya Maimaiti, John Q. Trojanowski, Virginia M.-Y. Lee, Kurt R. Brunden

**Affiliations:** 1 Center for Neurodegenerative Disease Research, Perelman School of Medicine, University of Pennsylvania, 3600 Spruce St, Philadelphia, PA 19104 USA; 2 Neurosience, Janssen Research & Development, Janssen Pharmaceutica NV (Division of Johnson & Johnson), Turnhoutseweg 30, 2340 Beerse, Belgium; 3 Discovery Sciences, Janssen Research & Development, Janssen Pharmaceutica NV (Division of Johnson & Johnson), Turnhoutseweg 30, 2340 Beerse, Belgium

**Keywords:** Alzheimer’s, Microglia, Pathology, Plaques, Tau

## Abstract

Dystrophic neuronal processes harboring neuritic plaque (NP) tau pathology are found in association with Aβ plaques in Alzheimer’s disease (AD) brain. Microglia are also in proximity to these plaques and microglial gene variants are known risk factors in AD, including loss-of-function variants of TREM2. We have further investigated the role of Aβ plaque-associated microglia in 5XFAD mice in which NP tau pathology forms after intracerebral injection of AD brain-derived pathologic tau (AD-tau), focusing on the consequences of reduced TREM2 expression and microglial depletion after treatment with the colony-stimulating factor 1 (CSFR1) inhibitor, PLX3397. Young 5XFAD mice treated with PLX3397 had a large reduction of brain microglia, including cortical plaque-associated microglia, with a significant reduction of Aβ plaque burden in the cortex. A corresponding decrease in cortical APP-positive dystrophic processes and NP tau pathology were observed after intracerebral AD-tau injection in the PLX3397-treated 5XFAD mice. Consistent with prior reports, 5XFAD × TREM2^−/−^ mice showed a significant reduction of plaque-associated microglial, whereas 5XFAD × TREM2^+/−^ mice had significantly more plaque-associated microglia than 5XFAD × TREM2^−/−^ mice. Nonetheless, AD-tau injected 5XFAD × TREM2^+/−^ mice showed greatly increased AT8-positive NP tau relative to 5XFAD × TREM2^+/+^ mice. Expression profiling revealed that 5XFAD × TREM2^+/−^ mice had a disease-associated microglial (DAM) gene expression profile in the brain that was generally intermediate between 5XFAD × TREM2^+/+^ and 5XFAD × TREM2^−/−^ mice. Microarray analysis revealed significant differences in cortical and hippocampal gene expression between AD-tau injected 5XFAD × TREM2^+/−^ and 5XFAD × TREM2^−/−^ mice, including pathways linked to microglial function. These data suggest there is not a simple correlation between the extent of microglia plaque interaction and plaque-associated neuritic damage. Moreover, the differences in gene expression and microglial phenotype between TREM2^+/−^ and TREM2^−/−^ mice suggest that the former may better model the single copy TREM2 variants associated with AD risk.

**Supplementary Information:**

The online version contains supplementary material available at 10.1186/s40478-021-01251-1.

## Introduction

The key pathological hallmarks of the AD brain are extracellular senile plaques comprising Aβ peptides and intracellular inclusions of misfolded tau protein, which normally binds microtubules (MTs) [9, 17]. Tau pathology can be found in Aβ plaque-associated neuronal processes as NP tau, within the soma as neurofibrillary tangles or in dendrites as neuropil threads. Aβ plaques develop first in the neocortex [5], whereas the initial distribution of tau pathology appears to be restricted to distinct brain areas including the locus coeruleus and trans-entorhinal cortex [3, 5] before progressing to the hippocampus and later to higher cortical regions. The “amyloid cascade” hypothesis [18, 41] suggests that cortical Aβ pathology initiates still poorly understood events that ultimately culminate in the cortical spread of tau pathology. Tau inclusions have been strongly implicated in the neurodegeneration of AD, as multiple immunohistochemical studies reveal a significant correlation between the degree of tau pathology and cognitive deficits in AD patients [1, 4, 48], a relationship that is not observed with Aβ plaque burden. Moreover, recent tau PET imaging studies further confirm a tight linkage between tau pathological burden and AD patient cognitive status [7, 42], as well as brain atrophy [45]. Thus, a key research objective is identifying mechanisms by which the formation of Aβ plaques promote the spread of neocortical tau pathology that is linked to AD dementia.

Plaque-associated neuronal processes may represent a key node in the amyloid cascade hypothesis, as this is a site where NP tau pathology forms in proximity to Aβ deposits. A model of coincident Aβ plaque and NP tau has recently been described [19] in which plaque-forming Tg mice (e.g., 5XFAD [31] or APP-knock-in [34]) develop widespread NP tau in the absence of tau overexpression after the intracerebral injection of a small amount of AD-tau. Endogenous mouse tau that accumulates within plaque-associated dystrophic processes [19], likely as a result of local disruption of MTs [11, 33, 50], is converted to insoluble NP tau aggregates that are recognized by multiple phospho-tau antibodies as well as antibodies to misfolded tau upon seeding by internalized AD-tau. Over time, this NP tau can lead to the subsequent formation of neurofibrillary tangles and neuropil thread tau pathology [19]. This unique model of combined Aβ and tau pathology provides a platform to investigate how Aβ pathology might affect the onset and spread of tau pathology.

When considering how Aβ plaques and/or oligomers might alter nearby neuronal processes, it is also important to consider the potential effects of plaque-associated microglia as found in both AD brain and in mouse models [6, 39]. A role of microglia in AD pathobiology is supported by a large body of data, including genome-wide association studies that have identified several microglial gene variants as risk factors for AD [29], including TREM2 variants [15, 23] that appear to affect microglial function [44]. Many studies have examined the potential influence of microglia in mouse models of Aβ plaque formation. Microglia can be quantitatively eliminated with CSF1R inhibitors [10], and whereas Aβ plaque burden was unchanged in older mice after CSF1R inhibitor treatment, there was a rescue of dendritic spines, improvement of cognitive function and reductions of neuron loss [32, 40]. More recent studies [37, 38] have examined the effect of depleting microglia from younger Aβ plaque-forming mice, where Aβ plaque burden was diminished after CSFR1 inhibitor treatment. Thus, microglia may play a role in the initial deposition of Aβ plaques, whereas they do not appear to affect later plaque development but nonetheless contribute to neuronal dysfunction.

As noted, TREM2 variants confer increased risk of AD with the R47H variant exerting the largest effect through a partial TREM2 loss-of-function with reduced binding of natural TREM2 ligands [2, 46, 51]. The R47H TREM2 substitution also causes a significant reduction of TREM2 protein expression in mice, although this is due to generation of a mis-spliced TREM2 transcript that is not formed in humans with the R47H allele [49]. There have been numerous studies [14, 22, 30, 46, 47, 52] investigating the consequences of TREM2 loss-of-function via genetic knockout (KO) in mouse models with Aβ pathology. Although there have been differences in the findings among these studies, particularly with regard to the effect of TREM2 KO on Aβ plaques [14, 22, 30, 46], there is an emerging consensus that TREM2 deficiency results in reduced microglial recruitment to and consequent containment of plaques, resulting in increased Aβ-mediated damage to nearby neuronal processes [13, 24]. Although TREM2 KO studies are informative, they may not fully model the heterozygous TREM2 variants linked to AD. There have been fewer studies examining plaque-forming mice harboring TREM2^+/−^ microglia, but it appears they exhibit some reduction in the number of plaque-associated microglia [43, 52] although less than that observed in TREM2^−/−^ mice. There may still be changes in plaque morphology in mice with TREM2 haploinsufficiency that leads to increased plaque-associated neuritic dystrophy [52], although a recent report [27] indicates that heterozygous TREM2 microglia show normal plaque interaction without the evidence of increased neuritic damage observed in TREM2^−/−^ plaque-bearing mice.

The existing literature suggests there are differences between the effects of microglial depletion and TREM2 knockout on early plaque deposition. In particular, whereas early depletion of microglia in 5XFAD mice appears to cause a lowering of plaque burden[37, 38] and plaque volume [38], TREM2 deficiency seems to have a more variable effect on plaque deposition that may depend on the mouse model. Moreover, the improved neuronal outcomes observed after microglial depletion in older plaque-bearing mice, where no changes in plaque burden were observed [32, 40], suggests that reducing microglial interaction with Aβ plaques can be beneficial. This contrasts with observations from TREM2 knockout studies, where reduced microglial plaque association results in enhanced neuritic damage.

Here, we have examined the consequences of both microglial depletion and TREM2 deficiency on the formation of NP tau pathology, utilizing our previously described model of concurrent Aβ plaque and NP tau pathology after intracerebral seeding with AD brain-derived tau [19]. Although a recent report has revealed that TREM2 KO, or expression of human R47H TREM2 on a mouse TREM2 null background, results in exacerbation of NP tau pathology after AD-tau injection of APP/PS1 mice [28], we extend these studies by comparing the effects of both TREM2^−/−^ and TREM2^−/+^ microglia in AD-tau-injected 5XFAD mice. In addition, we also assessed the effect of microglial depletion via CSFR1 inhibitor treatment. Depletion of microglia after CSFR1 inhibitor treatment in young 5XFAD mice resulted in decreased Aβ plaque burden and a corresponding reduction of NP tau pathology, including a trend toward reduced neuritic dystrophy in plaques that remained after microglial depletion that likely related to smaller plaque size. Moreover, although 5XFAD × TREM2^+/−^ mice had significantly more plaque-associated microglia than 5XFAD × TREM2^−/−^ mice, the amount of NP tau pathology was equal to or greater in 5XFAD × TREM2^+/−^ than 5XFAD × TREM2^−/−^ mice. Gene expression analyses revealed that 5XFAD × TREM2^+/−^ mice had DAM gene expression levels that were generally intermediate between 5XFAD × TREM2^+/+^ and 5XFAD × TREM2^−/−^ mice, and microarray expression profiling showed differences in gene ontology pathways, including those linked to microglial function, between 5XFAD × TREM2^+/−^ and 5XFAD × TREM2^−/−^ mice. Our data suggest that the amount of NP tau pathology observed in AD-tau-injected 5XFAD mice is not entirely predicted by the extent of microglial interaction with plaques, nor is development of appreciable tau pathology dependent on the complete loss of a DAM phenotype. The observed differences between TREM2 heterozygous and KO mice suggest that the former may better model the single copy TREM2 risk alleles in AD.

## Methods

*Mouse Breeding:* 5XFAD mice were kindly provided by Dr. Robert Vassar and were bred as previously described [31]. The generation of 5XFAD mice with differing TREM2 genotypes is summarized in Additional file 1: Fig. S1, resulting in 5XFAD^+/−^ × TREM2^+/+^, 5XFAD^+/−^ × TREM2^+/−^ and 5XFAD^+/−^ × TREM2^−/−^ mice on identical genetic backgrounds. The genotype of all mice was confirmed by PCR, with verification of the mutated APP and PS1 transgenes of 5XFAD mice as previously described [19], and TREM2 genotyping performed utilizing the following primers:

TREM2 common forward primer: TCA GGG AGT CAG TCA TTA ACC A.

TREM2 wild-type reverse primer: AGT GCT TCA AGG CGT CAT AAG T.

TREM2 KO reverse primer: CAA TAA GAC CTG GCA CAA GGA.

Expected TREM2 amplification products: KO = 396 bp; Wild type = 254 bp.

*Stereotaxic Injection of AD-Tau or Control Brain Extract:* AD brain-derived pathological tau and control brain extracts were prepared as previously described [16]. 5XFAD mice were injected with a total of 2 µg of AD-tau (typically 10–20% of total protein) or an equal volume of control brain extract in the hippocampus and overlying cortex of one hemisphere (2.5 µl of 0.4 mg/mg of AD-tau/site), at coordinates bregma: − 2.5 mm; lateral: 2 mm; depth: − 2.4 mm and − 1.4 mm from the skull, as previously described [16]. In one study, 5XFAD mice that had received either control or PLX3397-containing chow beginning at 1.5 months of age received stereotaxic injections at 3 months of age. There were 7 mice in each treatment group injected with AD-tau (Control chow, 4 males and 3 females; PLX3397 chow, 3 males and 4 females). In the control extract-injected 5XFAD mice, there were 6 mice in the PLX3397 and control treatment groups (3 males and 3 females). In another study, a total of 8 5XFAD mice (4 males and 4 females) from each TREM2 genotype received stereotaxic injections of AD-tau at 4 months of age. In both studies, mice survived an additional 3 months after intracerebral injections before sacrifice and post-mortem analyses.

*PLX3397 Dosing of 5XFAD mice:* PLX3397 (1Click Chemistry, Inc.) was formulated in chow at 1000 mg/kg or 290 mg/kg (Open Standard Diet; Research Diets, Inc.). Control chow was of the same composition but without added PLX3397. As summarized in Additional file 1: Fig. S2, groups of 1.5 month old 5XFAD mice received either control or 1000 mg/kg of PLX3397-containing chow for 1 week, after which the mice receiving PLX3397 were switched to chow containing 290 mg/kg of PLX3397. After 5 additional weeks on PLX3397 (290 mg/kg) or control chow, all mice received intracerebral injections of either AD-tau or control brain extract and were then maintained on control or PLX3397 (290 mg/kg) chow for an additional 3 months.

*Preparation of Brain Sections:* All study mice were sacrificed and perfused with phosphate-buffered saline (PBS) using protocols approved by the University of Pennsylvania IACUC. Brains were collected and fixed in formalin for 24 h at 4 °C, followed by transfer to 20% sucrose solution at 4 °C for 48 h. Coronal slices (30 mm) were obtained by using a brain mount, and these were cassetted and frozen in dry ice. A small hole was punctured on the left side of the midbrain to ensure proper identification of the hemisphere that had received AD-tau or control brain extract injections. Cassetted brain slices were stored at − 80 °C until they were sectioned (40 µm) and placed on slides, using a Thermo Shandon cryotome.

*Immunofluorescence (IF):* Frozen sections were stained with selected antibodies and fluorescently-labeled secondary antibodies to visualize microglia, phosphorylated tau, Aβ plaques, and APP positive dystrophic processes. Sections from 3 or 4 bregma levels from each study mouse were stained for analyses and utilized for quantification unless there was a technical flaw with a section, with a minimum of two evaluable bregma quantified from each mouse. The following antibodies and dyes were utilized: Aβ: NAB228 (1:20 K) monoclonal antibody (mouse anti-Aβ1-11; in-house); APP: 22C11 (1:1 K) mouse monoclonal antibody (APP N-terminus; Millipore, Inc.); microglia: Iba1 (1:1 K) rabbit monoclonal antibody (FujiFilm Wako Pure Chemical Industries, Ltd.); pTau: AT8 (1:1 K) mouse monoclonal antibody (tau pSer202/pThr205; Thermo-Fisher) and AT180 (0.33 µg/ml) mouse monoclonal antibody (tau pThr231; Thermo-Fisher); and misfolded tau: Alz50 (1:1000) monoclonal antibody (kind gift from Dr. Peter Davies). Sections were placed in wells of a 12-well plate containing 1 ml of Dulbecco’s phosphate-buffered saline (PBS), pH 7.4, with a maximum of 12 sections per well. Sections were washed (3 × 10 min) in PBS. In sections to be stained with 22C11 antibody, antigen retrieval was performed in citrate buffer (1:100 citric acid antigen unmasking solution; Vector Laboratories) for 1 h at 60 °C. All sections were blocked for 1 h at room temperature in 3% fetal bovine serum, 3% bovine serum albumin (w/v), 0.1% Triton X-100 in PBS solution. Primary antibodies were prepared in blocking buffer without Triton X-100 and incubated overnight at 4 °C, with the exception of 22C11 antibody which was incubated overnight at room temperature. Bound primary antibodies were detected with one of the following secondary antibodies: goat anti-mouse IgG2a conjugated with Alexa Fluor 647 (Thermo-Fisher); goat anti-mouse IgG1 conjugated with Alexa Fluor 488 (Thermo-Fisher); or goat anti-rabbit IgG conjugated with Alexa Fluor 568 (Millipore). A code was generated for all study mice to allow for masked processing of sections. Sections were imaged with a Nikon eclipse Ni microscope using either a 4X,10X, or 20X microscopic objective.

*Image Quantification:* Image J-Fiji software was used for all IF analyses in this study. Iba1-, AT8- and NAB228-stained sections within the cortex and subiculum were quantified by manually annotating each region of interest in 4X images after staining. Images for these analyses were obtained from bregma levels that captured the majority of tau pathology after AD-tau injection (bregma – 2.40, – 2.18, – 3.08, and – 3.52 for the PLX3397 study, and – 2.52, – 3.08, and – 2.70 for the TREM2 study). AT8 images were utilized from bregma – 2.40 in the PLX3397 study to avoid potential exogenous signal from AD-tau at the site of AD-tau injection. Images containing the injection site were not used for quantification. Once regions of interest were annotated, the image was converted to 8-bit and the remaining area outside of the annotated region was cleared. The image was converted to a grey scale and a threshold was applied to eliminate background signal, and integrated density (area × mean intensity) values were obtained. For visualization, the thresholded image was applied back to the original image to display the quantified IF signals (e.g., Additional file 1: Figs. S3 and S4). For NAB228 quantification, a particle size setting of 40-infinity was applied to exclude objects < 40 µM^2^. A particle size analysis (setting of 150-infinity) was utilized to quantify cortical plaque-associated APP at bregma levels – 2.70, – 2.46 and – 3.08 to avoid confounding signal from APP-positive neuronal cell bodies (see Fig. 5a). To closely examine cortical APP-positive dystrophic processes associated with plaques, higher power 20X images were obtained. Any APP-positive staining that was co-localized with or touching NAB228-positive plaques was annotated (see Fig. 6a). The manual selection was transferred to the 22C11 fluorescence channel, and the integrated density (area × mean density) values were obtained, which were normalized to the number of annotated plaques to give the APP integrated signal per plaque. In addition, the Aβ-positive plaque area was separately annotated in the NAB228 channel to allow calculation of the integrated Aβ signal. Similarly, to examine the plaque-associated cortical microglia, Iba1 staining that was co-localized with or touching NAB228-positive plaques was annotated in 20X images (Fig. 2b). The manual selection was transferred to the Iba1 fluorescence channel, and the Iba1 area values were obtained, which was normalized to the number of annotated plaques to give the relative Iba1 area per plaque.

*IF Statistics:* All IF quantifications were normalized to the appropriate control group (either control chow in the PLX3397 study or 5XFAD × TREM2^+/+^ in the TREM2 genotype study). As there are known differences between female and male 5XFAD mice (e.g., Aβ production and plaque load [31, 50]), IF values were normalized to the mean of the control group by sex. Statistical outliers within a treatment group (by sex) were identified by a two-sided Grubb’s or Dixon’s test and excluded from the analysis if p < 0.05. Statistical analyses with two study groups were done by a two-tailed unpaired T-test, and analyses of > 2 groups were done by one-way ANOVA with Tukey’s post-hoc comparisons. For the qPCR results, statistical analysis was done with a two-way ANOVA with Tukey's multiple comparisons test. All analyses were performed in GraphPad Prism. Error bars on all graphs represent the standard error of the mean.

*RNA extraction:* All study mice were sacrificed and perfused with PBS using protocols approved by the University of Pennsylvania IACUC. Brains were dissected, and the cortex and the hippocampus (with subiculum) from both the left and right hemisphere were collected, flash-frozen in liquid nitrogen and stored at − 80 °C until further use. Brains were obtained from 4 mice of genotype 5XFAD^+/−^ × TREM2^+/+^ (3 males and 1 female), 6 mice of genotype 5XFAD^+/−^ × TREM2^+/−^ (3 males and 3 females) and 6 mice of genotype 5XFAD^+/−^ × TREM2^−/−^ (3 males and 3 females). Tissues were transferred into Lysing Matrix D tubes (MP Biomedicals) and homogenized in 750 µl TRIzol™ reagent (Ambion) by using the MP Fast Prep24 homogenizer (MP Biomedicals) with 2 × 30 s at a speed of 6 m/s. Chloroform (150 µl) was added to the Lysing Matrix D tubes and thoroughly mixed with the homogenized samples. After centrifugation (15 min, 14,000 g, at 4 °C), 300 µl of the aqueous phase of each sample was transferred to a deep well plate and mixed with 300 µl of 70% ethanol. 600 µl of this mixed solution was transferred onto a RNeasy 96-well plate provided with the RNeasy 96-kit (Qiagen). The RNA was extracted according to the manufacturer’s instructions. RNA was eluted into 80 µl of RNase free water by means of two centrifugation steps (2 × 4 min, 6000 rpm at room temperature) and stored at − 80 °C until further use.

*cDNA conversion and Real-time qPCR of DAM gene mRNA:* The RNA concentration of the cortex and hippocampus samples from 5XFAD mice with different TREM2 genotypes was measured on the Nanodrop 8000 Spectrophotometer (Thermo Scientific). RNA (2 µg) was converted into cDNA (100 µl reaction volume) by using the High-Capacity cDNA reverse transcription kit (Thermo Fisher) and then diluted 1/9 into RNase-free water. For the real-time qPCR, 4.5 µl/well of diluted cDNA was combined with 0.5 µl/well of 20 × PrimeTime Std qPCR Taqman assay (IDT Integrated DNA Technologies) and 5.0 µl/well of Prime Time Gene Expression 2 × Master Mix with ROX reference dye (IDT Integrated DNA Technologies) into MicroAmp™ Optical 384-Well Reaction Plate with Barcode (Applied Biosystems). Reactions were evaluated on the QuantStudio 7 Pro Real-Time PCR system, using 384-well (Applied biosystems) default settings with an initial denaturation step at 95 °C for 10 min, followed by 40 cycles of 15 s at 95 °C for denaturation and 1 min at 60 °C for hybridization. For each assay all the samples were run in triplicates. The following 20 × PrimeTime Std qPCR Taqman assays were used:

**Table:** 

Assay target	Assay ID	Type[25]
*Trem2*	Mm.PT.58.7992121	DAM stage 2 marker
*Axl*	Mm.PT.58.11506780	DAM stage 2 marker
*Cst7*	Mm.PT.58.8810317	DAM stage 2 marker
*Lpl*	Mm.PT.58.46006099	DAM stage 2 marker
*Csf1*	Mm.PT.58.11661276	DAM stage 2 marker
*Ccl6*	Mm.PT.58.6955271	DAM stage 2 marker
*Itgax*	Mm.PT.58.42516719	DAM stage 2 marker
*Clec7a*	Mm.PT.58.42049707	DAM stage 2 marker
*Cx3cr1*	Mm.PT.58.17555544	DAM stage 1 marker
*P2ry12*	Mm.PT.58.43542033	DAM stage 1 marker
*Tmem119*	Mm.PT.58.6766267	DAM stage 1 marker
*Tyrobp*	Mm.PT.58.6069426	DAM stage 1 marker
*Ctsd*	Mm.PT.58.7639164	DAM stage 1 marker
*Apoe*	Mm.PT.58.33516165	DAM stage 1 marker
*B2m*	Mm.PT.39a.22214835	DAM stage 1 marker
*Tbp*	Mm.PT.39a.22214839	Reference gene
*Gapdh*	Mm.PT.39a.1	Reference gene
*Rplp0*	Mm.PT.58.43894205	Reference gene
*Polr2a*	Mm.PT.39a.22214849	Reference gene
*Gusb*	Mm.PT.39a.22214848	Reference gene
*Actb*	Mm.PT.39a.22214843.g	Reference gene
*Hprt*	Mm.PT.39a.22214828	Reference gene

The raw data files extracted from the QuantStudio 7 Pro Real-Time PCR system were uploaded into qBase + software version 2.4 (Biogazelle) for quality control and quantification of the Calibrated Normalized Relative Quantities (CNRQ). Only samples with at least two out of the three replicates with a replicate variability (difference in Cq) smaller than 0.5 were included. Reference genes used for normalization were determined by geNorm analysis. The optimal number of reference genes in our experimental setup was two (geNorm V < 0.15) and the most stable reference genes for both cortex samples and hippocampus samples were *Hprt* and *Gapdh*. Per tissue type scaling was done to the average of the control group 5xFAD Trem2 ^+ /+^ . For left and right hemisphere samples the CNRQ values were pooled per genotype group. CNRQ values were visualized using GraphPad Prism.

*Microarray analysis:* RNA from cortex and hippocampus was prepared as described above. Amplification and labelling of total RNA were performed using the GeneChip® PICO Reagent Kit following the manufacturer’s protocol (ThermoFisher 2016, P/N 902,790). Biotin-labeled target samples were hybridized to the GeneChip® Clariom S Mouse HT containing probes for over 20,000 genes. Target hybridization was processed on the GeneTitan® Instrument according to manufacturer’s instructions provided for Expression Array Plates (P/N 702,933). Images were analyzed using the GeneChip® Command Console Software (GCC) (ThermoFisher). Microarray data were processed using the statistical computing R-program (R version 3.6.1) and Bioconductor tools [12]. The gene expression values were normalized using Robust Multi-array Average (RMA) [21]. Individual probes were grouped into gene-specific probe sets based on Entrez Gene using the metadata package clariomsmousehtmmentrezg (version 22.0.0) [8].

## Results

### Depletion of microglia in young 5XFAD mice prior to intracerebral AD-tau seeding

Recent publications revealed that depletion of microglia from young 5XFAD mice with CSFR1 inhibitors led to a reduction of parenchymal Aβ plaques [37, 38], albeit with the extent of plaque lowering differing between studies. To investigate the consequences of microglial depletion on the formation of NP tau in 5XFAD mice, 1.5-month old 5XFAD mice that have not yet developed Aβ plaques [31] were treated with PLX3397 in chow for 1.5 months, followed by intracerebral injection of AD-tau. A similarly treated control group received chow without PLX3397, and additional groups received control or PLX3397-containing chow with injections of a control (non-diseased) brain extract. All groups then received control or PLX3397-containing chow for an additional 3 months prior to sacrifice (treatment paradigm summarized in Additional file 1: Fig. S2).

**Fig. 1:**
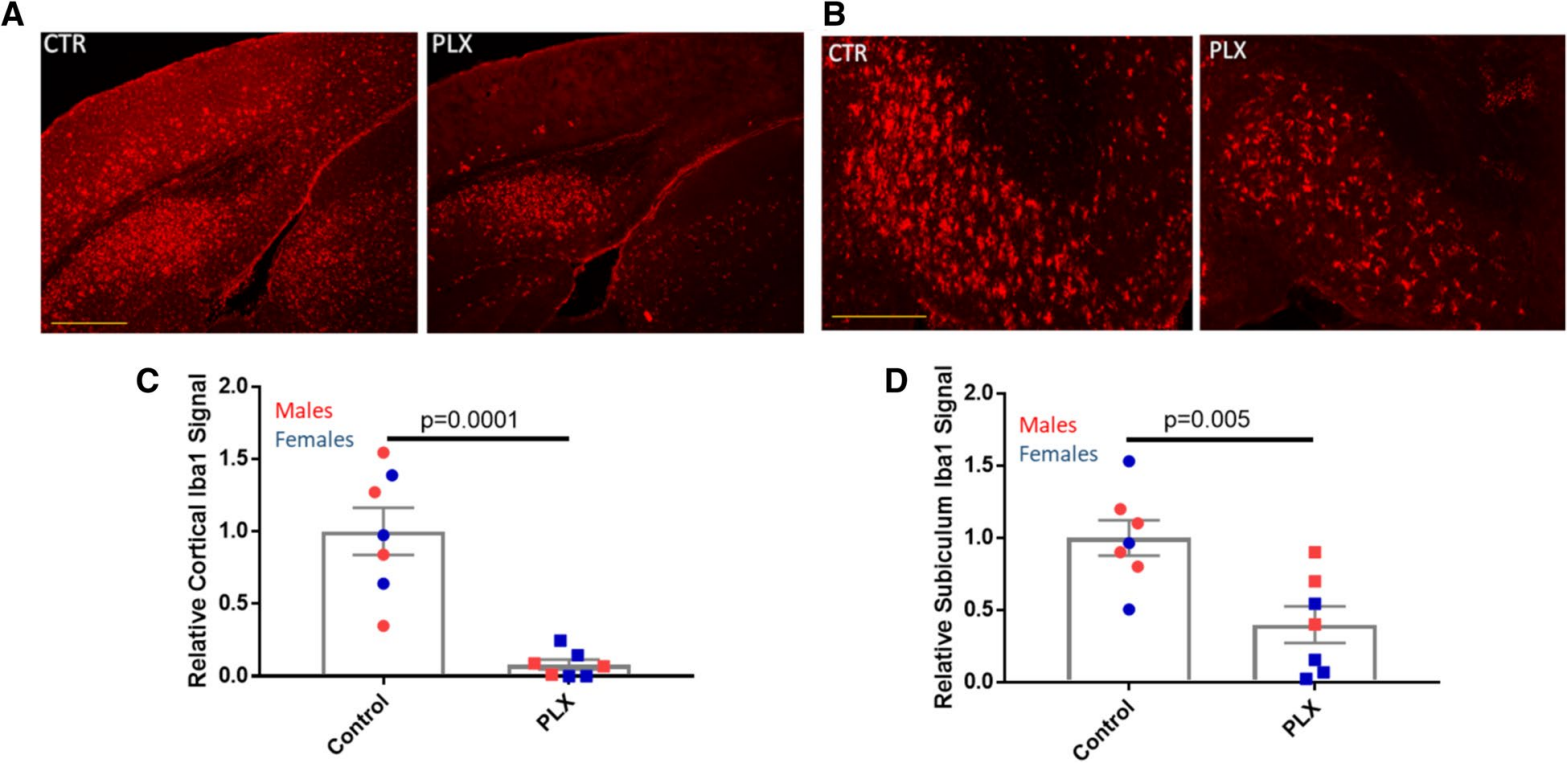
PLX3397 treatment caused significant reduction in brain microglia in AD-tau-injected 5XFAD mice. Iba1-positive microglia were greatly reduced in the cortex of PLX3397-treated 5XFAD mice (PLX) (**a**), as well as in the subiculum (**b**), relative to control mice (CTR), with quantification as shown in **c** and **d**, respectively. Scale bars represent 0.4 mm in A and 0.2 mm in B

**Fig. 2:**
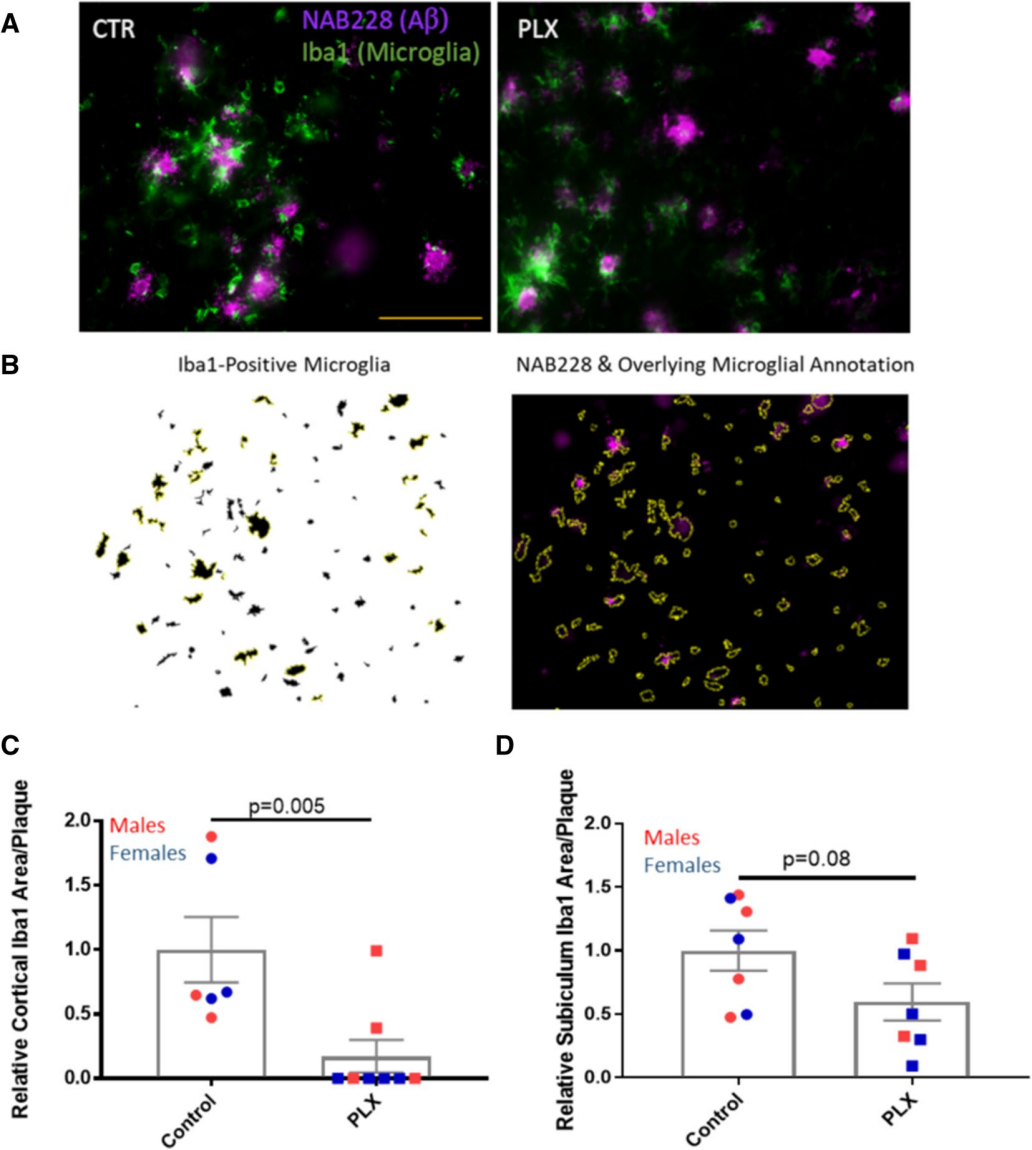
PLX3397 treatment led to a reduction of plaque-associated microglia in AD-tau-injected 5XFAD mice. **a** Plaque-associated microglia were reduced in the cortex of PLX3397-treated (PLX) compared to control (CTR) AD-tau-injected 5XFAD mice. **b** Example image of Iba1-positive microglia after thresholding (left), and subsequent overlaying of the thresholded image onto Aβ-stained plaques to identify plaque-associated microglia (right). Only the Iba1 signal that was plaque-associated was quantified, resulting in mean Iba1 area per plaque. Quantification of plaque-associated Iba1 area in the cortex (**c**) and subiculum (**d)**. Scale bar represents 50 µm

To confirm that PLX3397 treatment resulted in a significant ablation of brain microglia, frozen brain sections (40 µm) were stained with the microglial marker, Iba1. Quantification of integrated Iba1 IF signal from the AD-tau-injected 5XFAD mice (see Additional file 1: Fig. S3A) at 4 bregma levels revealed that those that had received PLX3397 had a 92% reduction of microglial staining in the cortex (Fig. 1a, c). A comparable reduction in cortical microglia was observed in PLX3397-treated 5XFAD mice that received intracerebral injections of control brain lysate (Additional file 1: Fig. S3B), confirming that the injection of AD-tau did not affect the extent of microglial depletion. A lesser but still highly significant microglial depletion was observed in the subiculum of PLX3397-treated 5XFAD mice (62%; Fig. 1b, d, S3A), a region of early plaque formation and high plaque density. We did not quantify results in the hippocampus, as 5XFAD mice at this age show less plaque deposition in this region than in the cortex and subiculum. These results are generally consistent with prior observations in 5XFAD mice [38, 40], where CSFR1 inhibitor treatment resulted in lesser depletion of microglia in areas with abundant Aβ plaques. To determine whether PLX3397 treatment also reduced microglia that were directly associated with plaques, we assessed Iba1-positive microglia that were adjacent to plaques as identified by dual IF staining of Iba1-positive microglia and Aβ plaques (NAB228 antibody; see Fig. 2a, b). Cortical plaque-associated microglia were quantified in the retrosplenial cortex since this is a region with relatively abundant and readily annotated Aβ plaques in the 5XFAD mice, and a significant 83% reduction of cortical plaque-associated microglia was observed in the PLX3397-treated mice (Fig. 2a, c). An analysis in the subiculum again revealed a less robust effect, with a 40% decrease in microglia surrounding plaques that did not reach statistical significance (Fig. 2d). In summary, treatment with the CSFR1 inhibitor led to large reductions of both non-plaque and plaque-associated microglia in the cortex of 5XFAD mice, and a lesser but meaningful reduction of microglia in the plaque-rich subiculum.

**Fig. 3:**
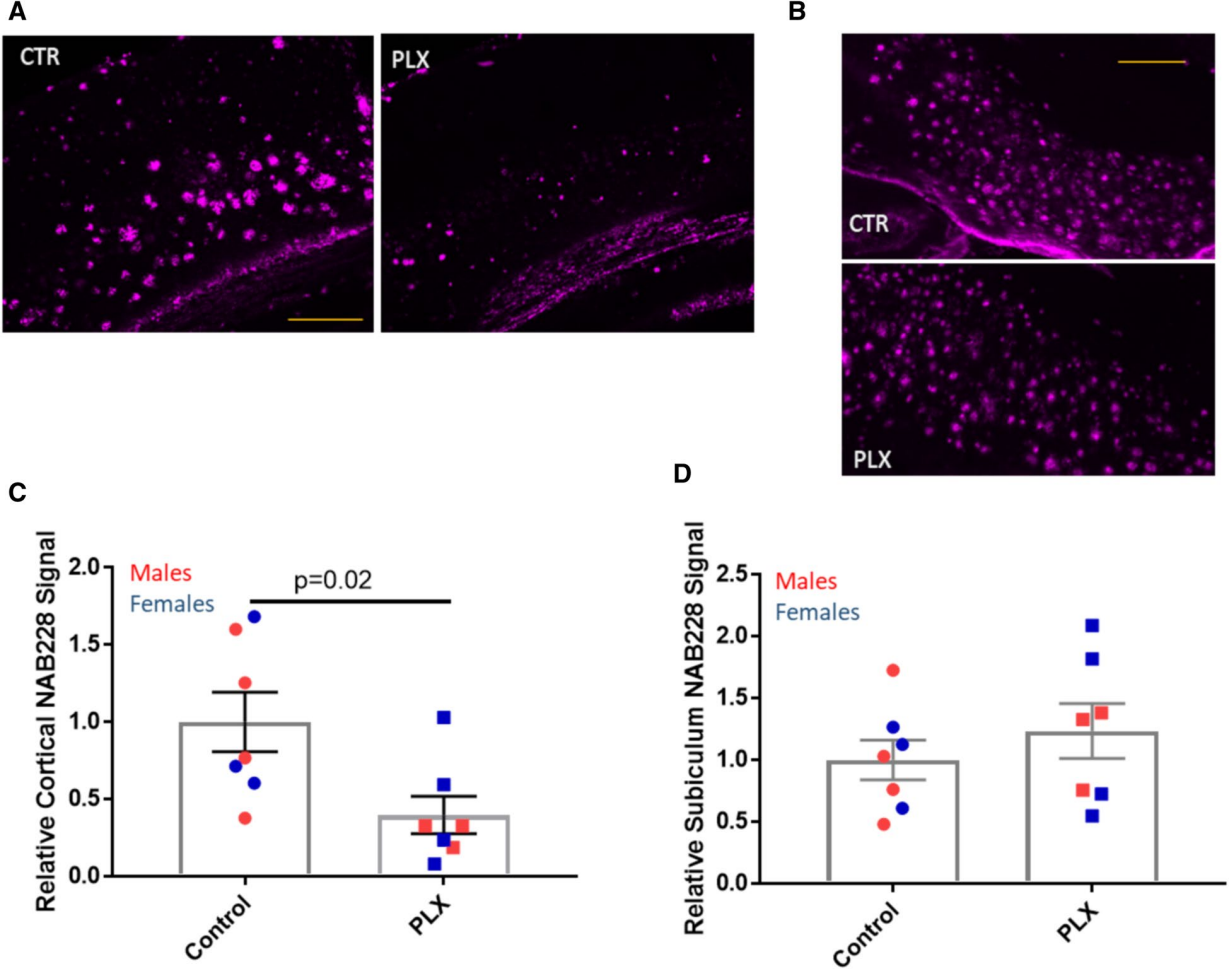
PLX3397 treatment reduced Aβ plaques in the cortex, but not subiculum. Representative images of NAB228-positive Aβ plaques in control (CTR) and PLX337-treated (PLX) AD-tau-injected 5XFAD mice are shown for (**a**) the cortex and (**b**) the subiculum, with quantification shown in (**c**) and (**d**), respectively. Scale bars represent 0.4 mm

Quantification of Aβ plaques in the brains of the AD-tau-injected 5XFAD mice (see example in Additional file 1: Fig. S4A) revealed a significant decrease in cortical Aβ plaque signal in the mice treated with PLX3397 (Fig. 3a, c). A similar reduction of cortical Aβ plaques was observed in the PLX3397-treated 5XFAD mice that were injected with control brain lysate, although this did not reach significance due to greater variability and smaller group sizes (Additional file 1: Fig. S4B). In contrast, there was no difference in plaque load in the subiculum of 5XFAD mice receiving PLX3397 compared to controls (Fig. 3b, d). These results are generally consistent with a recent report of young 5XFAD mice treated with PLX5622 [38], where plaque decreases were dependent on the extent of microglial depletion. Notably, we did not observe the large 90% plaque reduction throughout the brain as reported in another study with PLX3397-treated young 5XFAD mice [37].

**Fig. 4:**
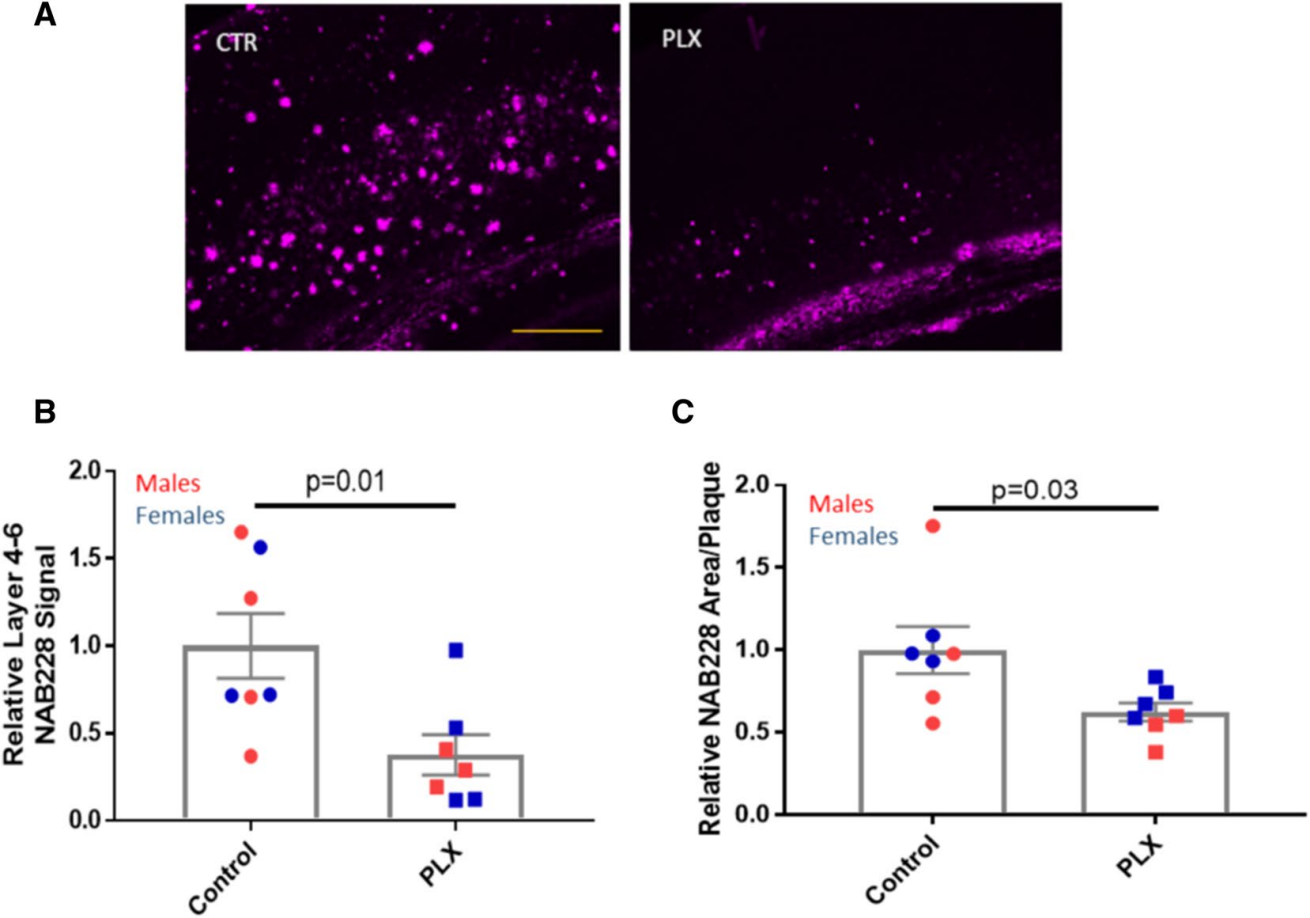
PLX3397 treatment reduced Aβ plaques and plaque size in layers 4–6 of the cortex. **a** Representative images of NAB228-positive Aβ IF in cortical layers 4–6 of control (CTR) and PLX3397-treated (PLX) AD-tau-injected 5XFAD mice. Quantification of total Aβ plaque signal in cortical layers 4–6 (**b**) and area per plaque in layers 4–6 (**c**). Scale bar represents 0.4 mm

**Fig. 5:**
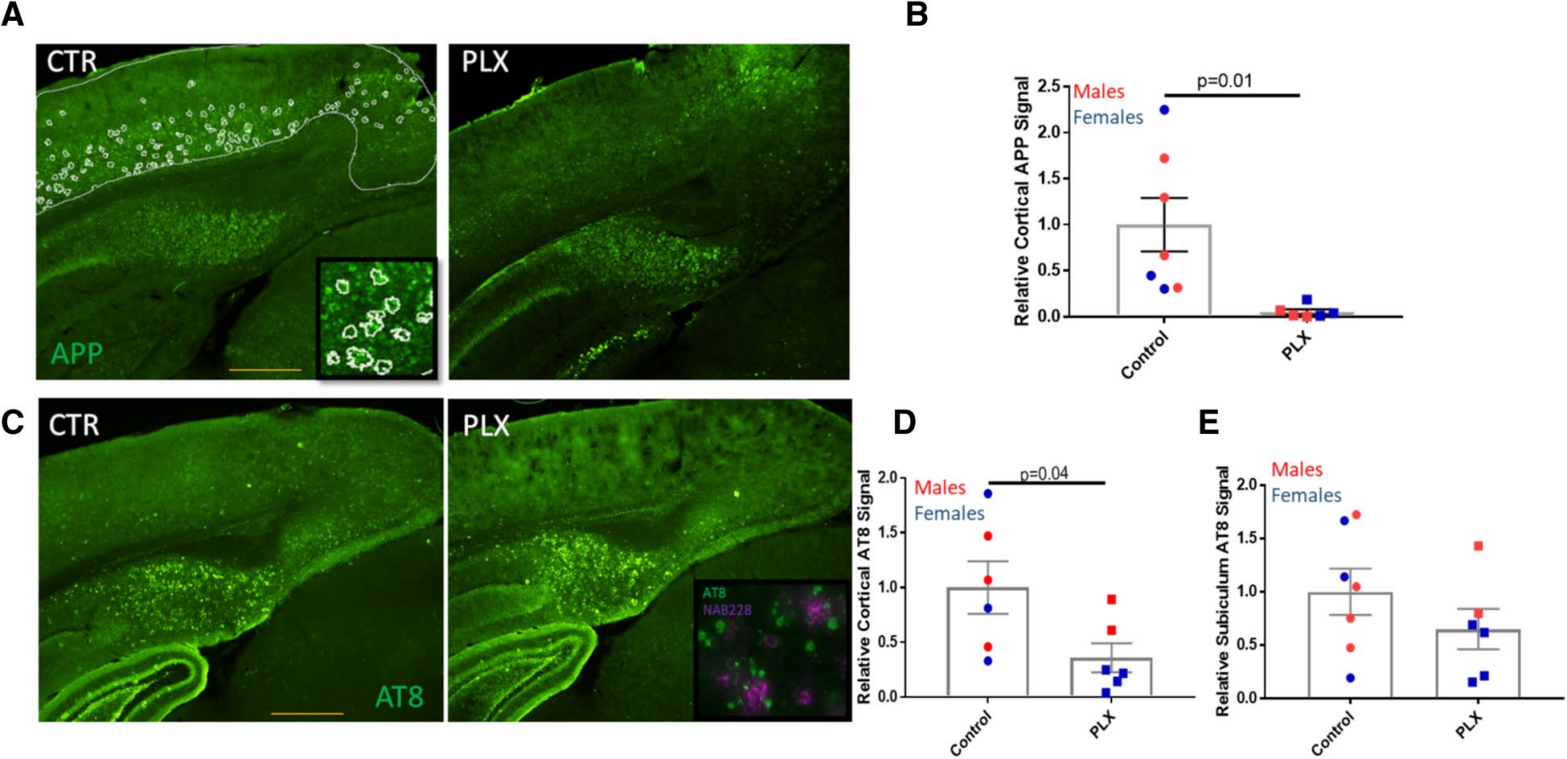
AD-tau-injected 5XFAD mice treated with PLX3397 had greatly reduced APP-positive dystrophic processes and AT8-positive NP tau. **a** Representative 22C11-positive APP IF images of a control (CTR) and PLX3397-treated (PLX) AD-tau-injected 5XFAD mouse, showing the annotation of the cortical region and selection of APP-positive dystrophic processes with exclusion of APP-positive neuronal soma (see insert). **b** Quantification of cortical APP-positive dystrophic processes. One female PLX3397-treated mouse was excluded from the analysis because useable brain sections were unavailable. **c** Representative images of AT8-positive tau in control (CTR) and PLX3397-treated (PLX) AD-tau-injected 5XFAD mice, with quantification of **d** cortical and **e** subiculum NP tau pathology. The inset in C is a dual-stained image from the subiculum showing that AT8 (green) staining is found in processes that are closely associated with Aβ plaques (NAB228; violet). Scale bars represent 0.4 mm

To further investigate the consequences of microglial depletion on plaques and plaque-associated neuronal processes, we focused on cortical layers 4–6, as the vast majority of cortical Aβ deposits were restricted to these layers in the 5XFAD mice. Not surprisingly, an analysis of total plaque load in these cortical layers mirrored the results observed from quantification of the entire cortex, with a significant 61% decrease in integrated Aβ plaque signal in the PLX3397 treatment group compared to the control group (Fig. 4a, b). An evaluation of the plaque size in layers 4–6 of the retrosplenial cortex, the cortical area with the greatest abundance of plaques, revealed that plaques that formed in the PLX3397-treated 5XFAD mice had a significantly smaller NAB228-positive area per plaque (Fig. 4c). These data are aligned with prior reports of decreased Aβ-immunoreactive plaque size in 5XFAD mice treated with PLX3397 [37, 38]. The overall decrease in cortical plaque burden in the PLX3397-treated 5XFAD mice suggests that a corresponding decrease in neuritic dystrophy might be observed, and this was indeed the case when APP-positive processes were quantified in the cortex (Fig. 5a, b). Prior studies demonstrated that soluble tau accumulates in the dystrophic processes around plaques [19], and thus the overall reduction of plaques and dystrophic neurites in the cortex led to a significant 64% decrease in the formation of AD-tau initiated AT8-positive cortical NP tau pathology (Fig. 5c, d). In contrast, there was a non-significant trend toward reduced AT8-positive tau in the subiculum upon microglial depletion (Fig. 5c, e), which likely reflects the unchanged Aβ plaque density in this area after PLX3397 treatment.

**Fig. 6:**
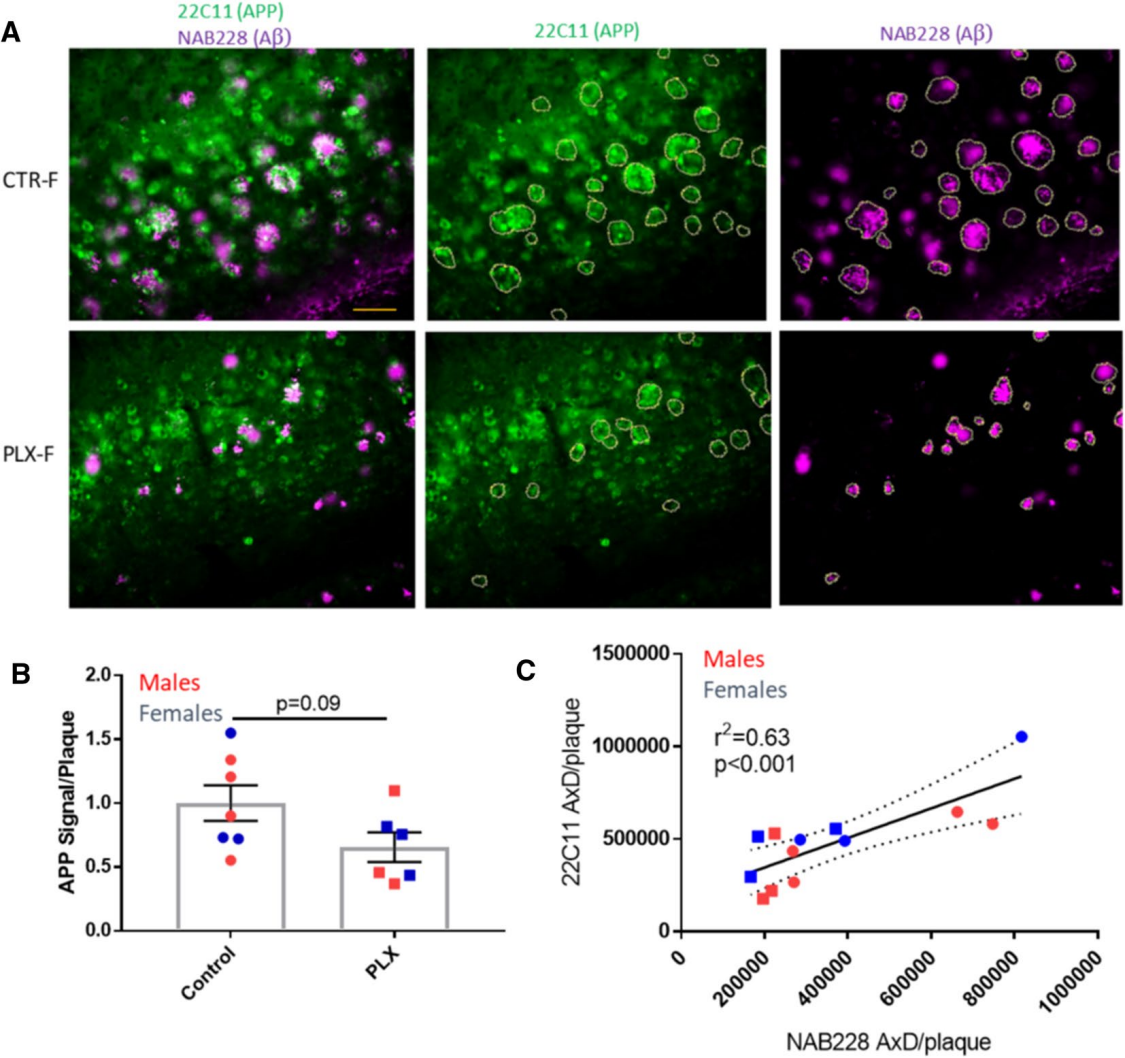
PLX3397 treatment of AD-tau-injected 5XFAD mice led to a trend toward less APP accumulation in plaque-associated processes. **a** Representative cortical images of NAB228 (Aβ) and 22C11 (APP) IF from control (CTR) and PLX3397-treated (PLX) female 5XFAD mice, with annotation of Aβ plaque-associated APP within the focal plane that were then quantified. **b** Quantification of Aβ plaque-associated APP signal per plaque. One PLX3397-treated female mouse was excluded from the analysis because brain sections were unavailable at all bregma levels. **c** Correlation between the mean integrated APP and Aβ signal per plaque for each study mouse shown in B. Circles = controls; Squares = PLX3397-treated. Dashed line represents the 95% confidence limits. Scale bar represents 50 µm

Although there was an overall reduction of cortical neuritic dystrophy in the PLX3397-treated 5XFAD mice that corresponded with decreased plaque burden, a remaining question was whether reduced microglial plaque interaction (Fig. 2c) resulted in greater neuritic dystrophy in those cortical plaques that did form in the PLX3397-treated mice. To investigate this, we measured APP signal in the area immediately adjacent to plaques in cortical layers 4–6. Interestingly, the integrated APP value per plaque showed a trend toward reduction in PLX3397-treated 5XFAD mice compared to the control 5XFAD mice (Fig. 6a, b). As a reduction in cortical Aβ plaque size was also observed in the PLX3397 treatment group, we assessed whether the extent of neuritic damage around remaining plaques in the PLX3397-treated mice was related to plaque size. Indeed, a significant correlation was observed between the mean integrated APP and Aβ signals per plaque from sections of both control and PLX3397-treated 5XFAD mice (Fig. 6c). Notably, it appears that reduced microglial clustering around cortical plaques in the PLX3397-treated 5XFAD mice did not result in increased neuritic damage, which instead seemed to be dependent on plaque size.

### Intracerebral AD-tau seeding of 5XFAD mice with differing TREM2 genotypes

The results from our PLX3397 microglial depletion studies support recent observations of microglia playing a role in initial Aβ plaque deposition [20, 37, 38]. The reduced cortical Aβ plaque burden found after microglial elimination led to an overall decrease in cortical plaque-associated APP- and AT8-positive dystrophic processes in AD-tau-injected 5XFAD mice. To further investigate the relationship between microglia and NP tau pathology, we conducted studies in which 5XFAD × TREM2^−/−^, 5XFAD × TREM2^+/−^ and 5XFAD × TREM2^+/+^ mice on identical genetic backgrounds (Additional file 1: Fig. S1) received AD-tau injections. Groups of mice (males and females) of each TREM2 genotype received intracerebral injections of AD-tau at 4-months of age when Aβ plaques were abundant, followed by an evaluation of brain pathological endpoints 3 months later.

**Fig. 7:**
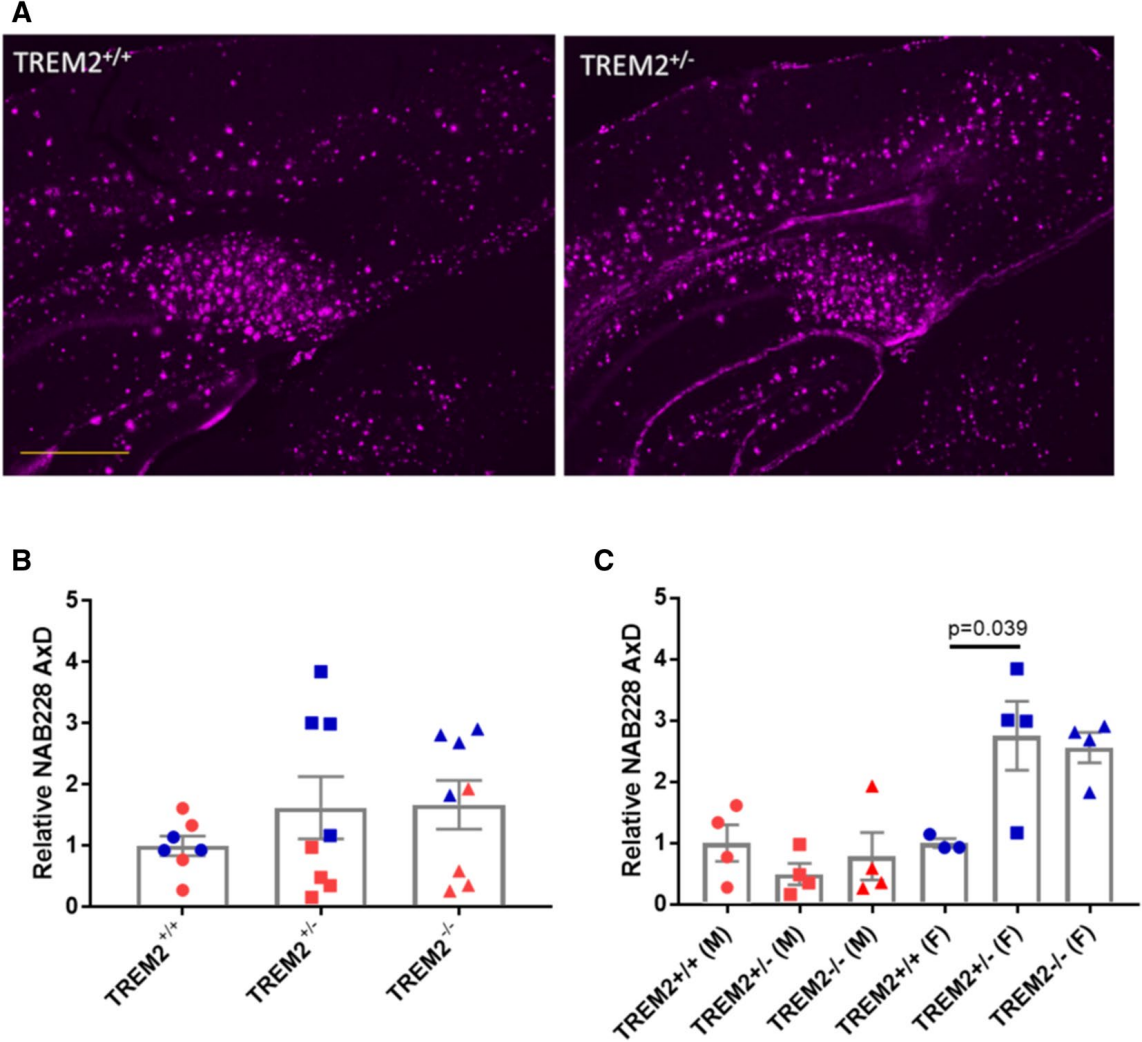
Aβ plaque burden was differentially affected by TREM2 genotype in male and female AD-tau-injected 5XFAD mice. **a** Representative image of cortical and subiculum NAB228-positive Aβ plaques in female 5XFAD × TREM2^+/+^ and 5XFAD × TREM2^+/−^ mice. **b** Quantification of cortical Aβ plaque burden in AD-tau-injected 5XFAD mice as a function of TREM2 genotype. **c** Cortical Aβ plaque burden graphed by sex for each TREM2 genotype. Scale bar represents 0.4 mm

Aβ plaques were quantified in the cortex of all three 5XFAD × TREM2 genotypes. Although a difference in overall cortical plaque load was not observed across groups when all mice were included (Fig. 7a, b), we did note an increase in Aβ plaques in female 5XFAD × TREM2^−/−^ and 5XFAD × TREM2^+/−^ mice compared to female 5XFAD × TREM2^+/+^ mice, which reached significance for the heterozygous TREM2 mice (Fig. 7c). In contrast, there was a trend toward reduced plaque load in the male TREM2 heterozygous and KO mice relative to male 5XFAD × TREM2^+/+^ mice (Fig. 7c). The cause of these sex-dependent differences is unclear, although it may relate to the greater Aβ production in young female than male 5XFAD mice [31, 50]. Interestingly, it appeared that changes in plaque burden by sex were comparable between the 5XFAD × TREM2^−/−^ and 5XFAD × TREM2^+/−^ mice.

**Fig. 8:**
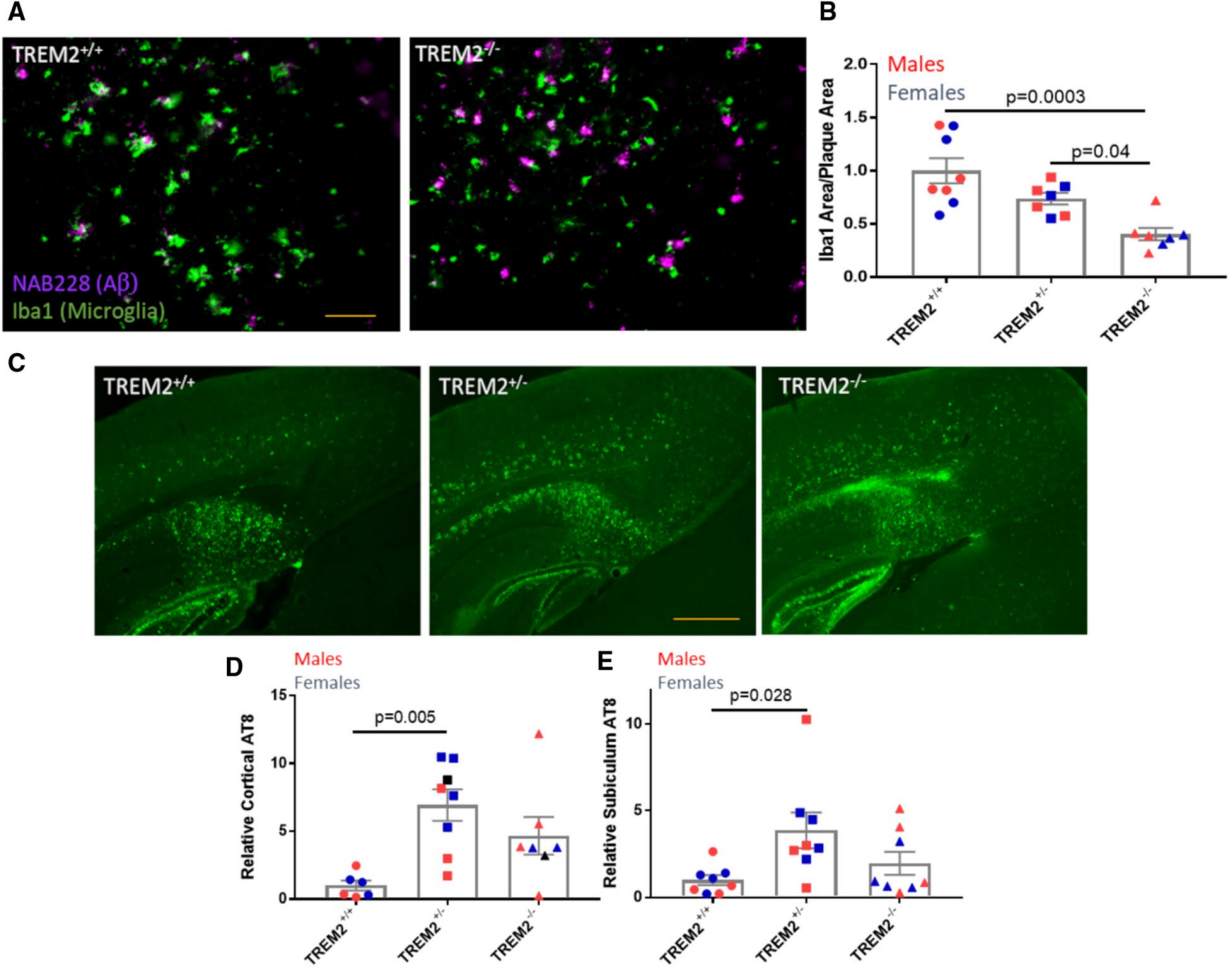
AD-tau-injected 5XFAD × TREM2^+/−^ mice had greater Aβ plaque-associated microglia than 5XFAD × TREM2^−/−^ mice but comparable AT8-positive NP tau pathology **a** Representative images of cortical Aβ plaque-associated microglia in 5XFAD × TREM2^+/+^ and 5XFAD × TREM2^−/−^ mice. **b** Quantification of cortical Aβ plaque-associated microglia area per plaque as a function of TREM2 genotype. **c** Representative images of AT8-positive NP tau pathology in the cortex and subiculum of female 5XFAD mice with differing TREM2 genotype. Quantification of **d** cortical and **e** subiculum AT8 NP tau pathology as a function of TREM2 genotype. Scale bars represent 50 µM in A and 0.4 mm in B

Several studies have indicated that TREM2 knockout leads to reduced microglial clustering around plaques, resulting in plaques of a more diffuse character that appear to be detrimental to nearby neuronal processes. An assessment of Iba1-positive microglial association with cortical Aβ plaques in the 5XFAD mice with differing TREM2 genotypes replicated these prior observations, with a significant decrease in Iba1 area per plaque in the 5XFAD × TREM2^−/−^ mice compared to the to 5XFAD × TREM2^+/+^ mice (Fig. 8a, b). The 5XFAD × TREM2^+/−^ mice showed a smaller, non-significant reduction of plaque-associated microglial area when compared to 5XFAD × TREM2^+/+^ mice (Fig. 8b), with significantly more plaque-associated microglial area than 5XFAD × TREM2^−/−^ mice. The smaller reduction of plaque-associated microglia in 5XFAD × TREM2^+/−^ than 5XFAD × TREM2^−/−^ mice is consistent with a prior report [52], and a more recent study reported no change in plaque-associated microglia in TREM2^+/−^ mice relative to TREM2^+/+^ mice [27]. There did not appear to be sex-dependent differences in microglial association with plaques in any of the 5XFAD × TREM2 genotypes, suggesting that the observed differences in Aβ plaque burden by sex were not the result of differing microglial interaction with plaques. In this regard, it is interesting that a decrease in cortical plaque-associated microglia in TREM2^−/−^ mice resulted in increased Aβ plaques in female 5XFAD mice and a non-significant change when both sexes are combined, whereas microglia depletion in the PLX3397-treated 5XFAD mice led to decreased cortical Aβ plaques (Fig. 2a, c). This differing outcome could relate to the fact that TREM2 KO does not result in the loss of parenchymal microglia that may contribute to Aβ plaque biogenesis [37, 38], as occurs upon PLX3397 treatment.

As a leading hypothesis is that reduced microglial interaction with plaques in TREM2 KO mice leads to greater Aβ-mediated damage to nearby processes, we assessed the extent of cortical NP AT8-positive tau pathology in the 5XFAD mice with differing TREM2 genotype. Indeed, we observed increases of plaque-associated AT8 tau pathology in both 5XFAD × TREM2^−/−^ and 5XFAD × TREM2^+/−^ mice relative to the 5XFAD mice with normal TREM2 expression (Fig. 8c), with the latter reaching statistical significance in both the cortex (Fig. 8d) and the subiculum (Fig. 8e), which had the greatest amount of AT8 pathology in the AD-tau-injected 5XFAD mice. The increase in cortical NP AT8 in the 5XFAD × TREM2^+/−^ mice was greater in females than males, consistent with the greater cortical Aβ plaque burden in the female 5XFAD × TREM2^+/−^ mice (Fig. 7c). A sex difference in cortical AT8 staining was not evident in the 5XFAD × TREM2^−/−^ mice, perhaps due to greater variability in the male AT8 group. Interestingly, there was a non-significant trend toward greater AT8-positive NP pathology in the 5XFAD × TREM2^+/−^ mice than the 5XFAD × TREM2^−/−^ mice, even though the latter had significantly greater depletion of plaque-associated microglia. This suggests that the extent of neuritic dystrophy and NP tau pathology is not entirely correlated with the extent of microglial interaction with plaques, as was also observed from the CSFR1 inhibitor studies described above. Immunostaining of sections from 5XFAD × TREM2^+/−^ mice with the AT180 (p231 Tau) and Alz50 (misfolded tau) antibodies (Additional file 1: Fig. S5) further confirmed that the AD-tau-seeded dystrophic processes in these mice harbor pathologic tau.

**Fig. 9:**
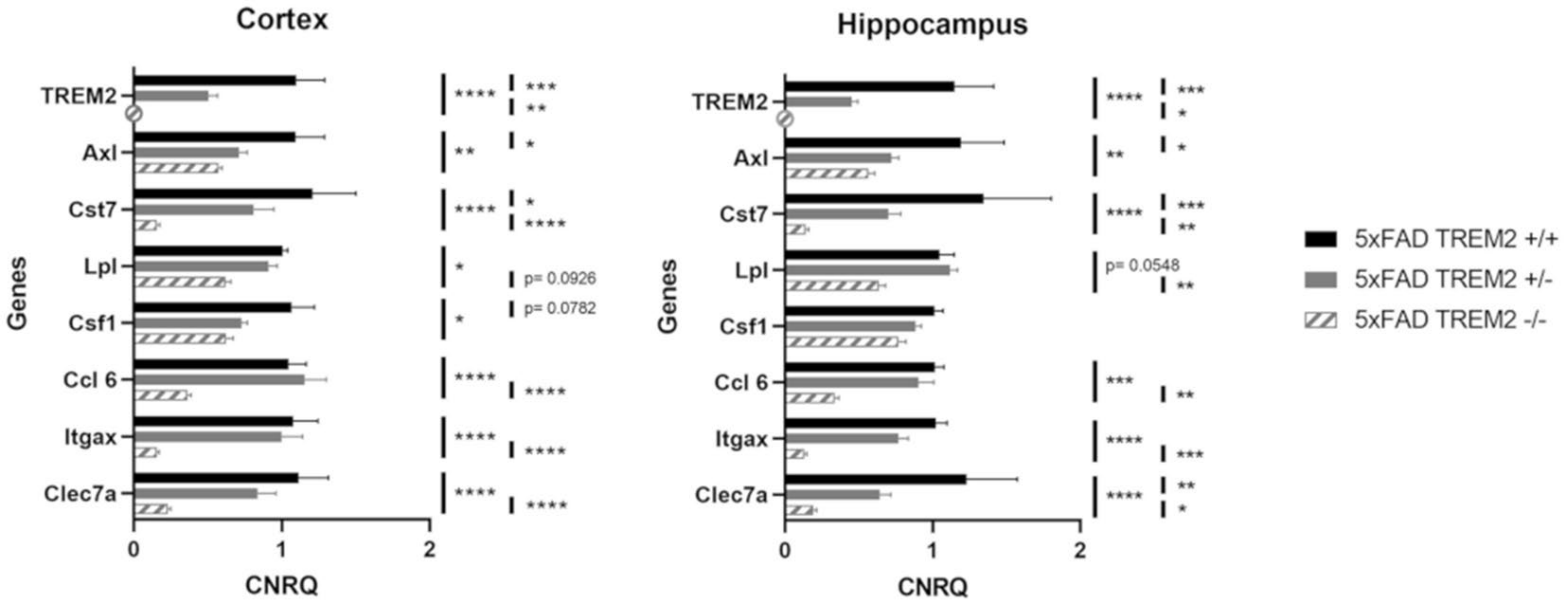
Reduced TREM2 expression led to reduced mRNA expression of DAM stage 2 markers in AD-tau-injected 5XFAD mice. qPCR analysis of DAM stage 2 genes in cortex and hippocampus samples of AD-tau-injected 5XFAD × TREM2^+/+^, 5XFAD × TREM2^+/−^ and 5XFAD × TREM2^−/−^ mice. Data from male (n = 3) and female (n = 3, except n = 1 for 5XFAD × TREM2^+/+^) mice were pooled, and samples of left and right hemisphere from each mouse were used as individual data points (data by sex and hemisphere can be found in Additional file 1: Fig. S6). TREM2 was below the threshold of detection in the 5XFAD × TREM2^−/−^ mice and thus expression is shown as zero. *p < 0.05, **p < 0.01, ***p < 0.001, ****p < 0.0001

Plaque-associated microglia have been shown to have an altered phenotype with changes in expression of several key genes and have been referred to as DAM [25] or microglia with a neurodegenerative phenotype (MGnD) [26]. DAM and MGnD gene expression depends on the TREM2 pathway [25, 26], and thus TREM2 KO mice lose the classical DAM expression profile (referred to as DAM stage 2) and acquire an intermediate phenotype denoted as DAM stage 1 [25]. As extensive NP tau pathology was found in 5XFAD × TREM2^+/−^ mice even though they had significantly greater microglial plaque association than TREM^−/−^ microglia, we analyzed brain DAM gene expression in the AD-tau injected 5XFAD mice with differing TREM2 genotypes. By combining both male and female mice within each genotype to simplify the analyses, stage 2 DAM genes were as expected decreased in both the cortex and hippocampus of the 5XFAD × TREM2^−/−^ mice compared to the 5XFAD × TREM2^+/+^ mice (Fig. 9; data by sex and hemisphere can be found in Additional file 1: Fig. S6). Stage 1 DAM genes were decreased to a limited extent as well in both the cortex and hippocampus (Additional file 1: Fig. S7; data by sex and hemisphere can be found in Additional file 1: Fig. S8). The stage 2 DAM gene expression profile in the 5XFAD × TREM2^+/−^ mice revealed levels that were generally intermediate between the TREM2^+/+^ and TREM2^−/−^ 5XFAD mice. Thus, these data suggest that enhancement of NP tau pathology does not depend on full reduction of Stage 2 DAM gene expression. In contrast, a significant decrease of microglial plaque association may depend on a large reduction in Stage 2 DAM gene expression since the TREM2^+/−^ microglia maintained appreciable plaque interaction (Fig. 8b).

**Fig. 10:**
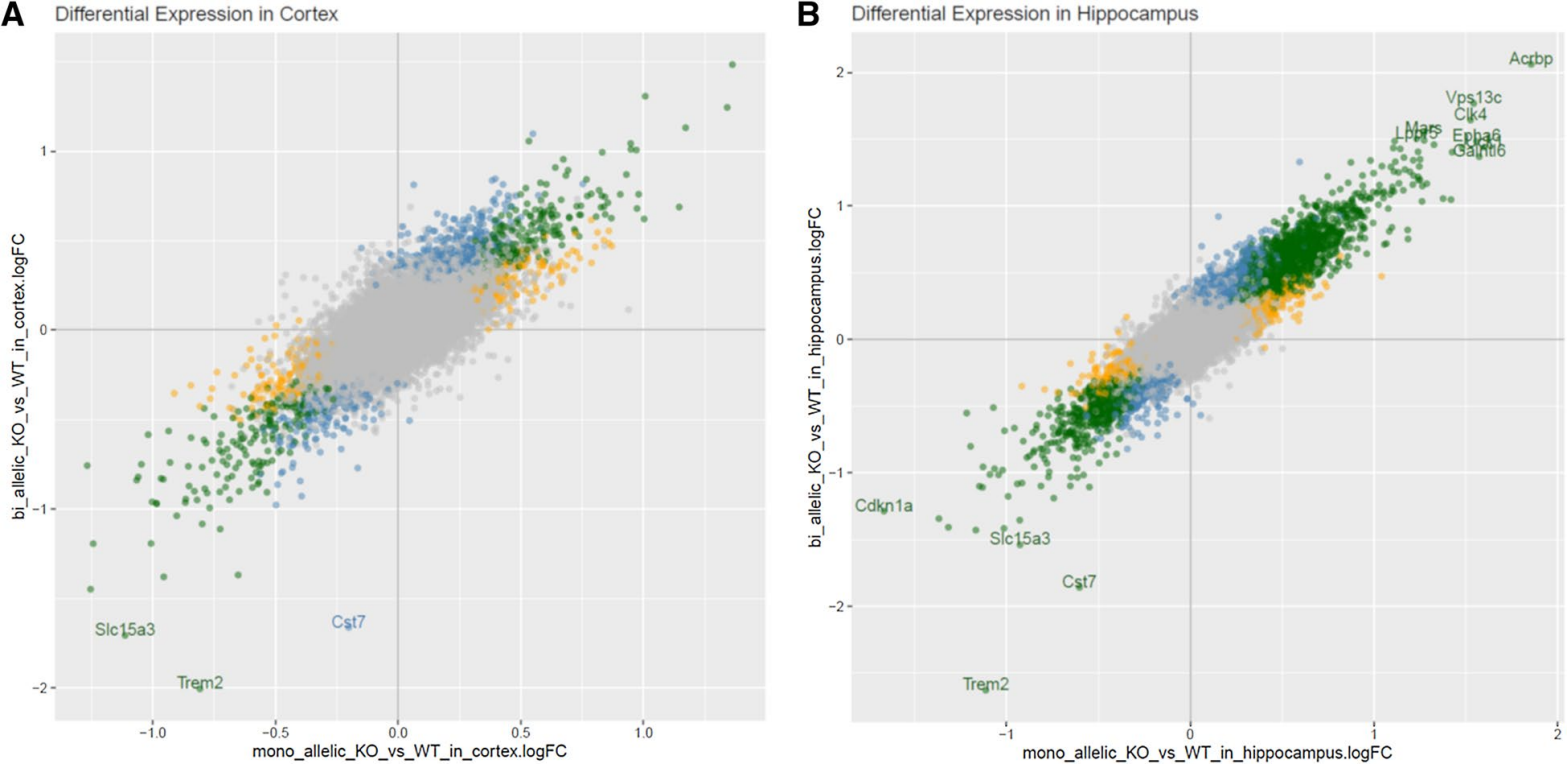
Comparison of the magnitude of differential gene expression between AD-tau injected 5XFAD × TREM2^+/+^ (WT) mice and 5XFAD × TREM2^−/−^ (bi-allelic KO) or 5XFAD × TREM2^+/−^ (mono-allelic KO) mice in the cortex (**a**) and hippocampus (**b**). Genes with an absolute logFC > 1.5 are annotated. Grey = non-significant difference; Yellow = Significant difference between TREM^+/+^ and TREM2^+/−^; Blue = Significant difference between TREM^+/+^ and TREM2^−/−^; Green = Significant in both previous comparisons

To further compare the AD-tau injected 5XFAD × TREM2^−/−^ and 5XFAD × TREM2^+/−^ mice, microarray analyses were conducted on mRNA isolated from the cortex and hippocampus of the study mice. As expected, mRNA levels were significantly changed for many genes when comparing the different TREM2 genotypes, with the largest number of changes occurring between the 5XFAD x TREM2^+/+^ mice and those lacking one or both copies of TREM2 (Additional file 1: Table S1). Interestingly, whereas partial or complete TREM2 deficiency led to many shared gene expression changes relative to TREM2-sufficient 5XFAD mice, there were also many genes where expression was uniquely significant in the TREM2 heterozygous or TREM2 KO mice relative to 5XFAD × TREM2^+/+^ mice (Fig. 10). A direct comparison of gene expression between the 5XFAD × TREM2^+/−^ and 5XFAD × TREM2^−/−^ mice revealed a number of significant changes in both the hippocampus and cortex (Additional file 1: Table S1). A gene ontology analysis of biological processes (Additional file 1: Table S2) indicated multiple pathways related to macrophage/microglial function and activation that were different between the AD-tau injected 5XFAD TREM2 heterozygous and TREM KO mice (only pathways with p-value of  < 1 × 10^–6^ are shown). In addition, several pathways related to cellular respiration and mitochondrial function also differed between these TREM2 genotypes. As the microarray analyses assessed changes within all cell types in the brain, it is unclear whether these latter changes are in neurons and/or glia. A comparison of the gene expression differences between the 5XFAD × TREM2^−/−^ and 5XFAD × TREM2^+/−^ mice in the cortex and hippocampus are summarized in Additional file 1: Fig. S9, with the most significant changes being in TREM2 and the DAM stage 2 gene, Cst7 (top hits in cortex and hippocampus summarized in Additional file 1: Table S3). The findings of microglial-related pathway and DAM gene expression differences between the 5XFAD × TREM2^−/−^ and 5XFAD × TREM2^+/−^ mice, combined with differing microglial plaque-association but similar NP tau pathology after AD-tau injection, emphasize that TREM2 heterozygous and KO mice are distinct and that the former may better model the heterozygous TREM2 variants linked to AD risk.

## Discussion

The genetic linkage of microglial TREM2 loss-of-function variants with increased risk of AD has led to numerous studies [14, 22, 30, 46, 47, 52] characterizing the consequences of reduced TREM2 expression on Aβ pathology and plaque-associated neuritic damage. There have been differences in the findings among these studies, particularly with regard to the effect of TREM2 KO on Aβ plaques, but a general consensus has emerged that TREM2 deficiency results in reduced microglial containment of plaques, leading to increased Aβ-mediated damage to nearby neuronal processes [13, 24]. The differing reports on the effect of TREM2 deficiency on Aβ plaque burden may be due to the use of differing mouse models and mice of different ages.

Although there is experimental support for the hypothesis that reduced microglial interaction with Aβ plaques in TREM2 KO mice leads to increased damage to nearby neuronal processes, reports of microglial depletion with CSFR1 inhibitors in Aβ plaque-forming mice suggest that reduced microglial containment of plaques is not always harmful to neurons. For example, treatment of 10-month old 5XFAD mice [31] with the CSFR1 inhibitor, PLX3397, for one-month resulted in the elimination of ~ 80% of microglia [40] and although this treatment did not affect Aβ plaque levels, it did rescue dendritic spines and reduce neuronal loss in the subiculum. This suggests a detrimental role of microglia on neuronal function that is independent of effects on Aβ plaques and that a reduction in plaque- (and non-plaque)-associated microglia can be beneficial. This conclusion is further support by a report with another CSFR1 inhibitor in APP/PS1 transgenic mice, where dosing from 6- to 9-months of age led to improvements in cognitive function without significant changes in Aβ plaque load [32].

We have further investigated the consequences of reduced microglial interaction with Aβ plaques utilizing our previously described model of Aβ plaque and NP tau pathology that is facilitated by intracerebral seeding of 5XFAD mice with AD brain-derived tau [19]. Initial studies assessed the consequences of microglial depletion in this model utilizing PLX3397, with dosing initiated at 1.5 months of age when Aβ plaques have not yet formed in the brains of the 5XFAD mice. Dosing continued until 3 months of age, when the mice received intracerebral injection of AD-tau, with PLX3397 exposure maintained until the mice reached 6 months of age. As expected, a large reduction of microglia was observed in the brain, including plaque-associated microglia that was particularly evident in the cortex. In agreement with recent studies in young 5XFAD mice [37, 38], treatment with the CSFR1 inhibitor led to a reduction in Aβ plaques that was significant in the cortex but not in the subiculum. Moreover, plaques that did form in the PLX3397-treated 5XFAD cortex had a smaller overall size. The reduction of cortical plaque burden in the 5XFAD mice treated with PLX3397 led to a decrease in plaque-associated APP-positive dystrophic processes, with a corresponding reduction of AT8-positive NP pathology. Moreover, there was a trend toward decreased neuritic dystrophy in proximity to remaining cortical plaques in the CSFR1 inhibitor-treated mice, with a significant correlation between the amount of plaque Aβ and neuritic dystrophy in both PLX3397- and vehicle-treated 5XFAD mice. This suggests that the degree of neuritic damage was driven by the amount of plaque Aβ irrespective of the extent of microglial-plaque interaction.

In contrast to CSFR1 inhibitor-mediated microglial ablation, neither partial or complete reduction of TREM2 expression led to significant changes in Aβ plaque burden in combined groups of male and female 5XFAD mice, although we did note sex-dependent differences in which TREM2 deficiency resulted in increased plaque burden in female 7-month old 5XFAD mice and a trend toward reduced plaque load in male 5XFAD mice. Although prior studies [22, 46] have examined the effect of TREM2 KO on Aβ plaque formation, with evidence of increased plaque burden in older 5XFAD × TREM2 KO mice relative to control 5XFAD mice, these studies did not differentiate male and female mice. However, a recent study [30] found results similar to ours in that plaque load was significantly increased in 6–7- month old female PS2APP × TREM2^−/−^ mice compared to TREM2-sufficient PS2APP mice, whereas this change was not observed in the corresponding male mice. The authors did not speculate on the cause of this sex-dependent difference and we are unsure of the mechanism leading to these differences, although it may possibly relate to greater Aβ production by young female than male 5XFAD mice [31, 50]. For example, if TREM2 deficiency affects microglial phagocytosis or catabolism of Aβ, then elevated Aβ release by young female 5XFAD mice may result in enhanced plaque deposition when microglia are TREM2-deficient, whereas this would not be as apparent in young male 5XFAD mice with lower basal Aβ release. However, this is only a hypothesis and further investigation would be required to gain a better understanding of these observations.

A notable difference between CSFR1 inhibitor treatment and TREM2 deficiency is that the former depletes brain microglia, whereas loss of TREM2 affects microglial phenotype. Thus, TREM2 heterozygous or KO microglia may still contribute to the production of Aβ plaques and plaque-associated neuritic damage, and our data suggest that decreased TREM2 expression may in fact facilitate plaque development in female 5XFAD mice. Notably, a very recent publication reveals that microglial phagocytosis of Aβ promotes the formation of mature plaques, and that KO of the TAM receptor kinases Axl and Mer results in fewer plaques in APP/PS1 mice [20]. This group showed that TREM2^−/−^ microglial are capable of greater Aβ phagocytosis than the Axl and Mer KO mice, suggesting that they could still contribute to plaque biogenesis. However, the ability of TREM2-deficient microglia to contribute to plaque formation may wane with age, as another recent study revealed that anti-sense oligonucleotide knockdown of TREM2 expression in 10-month old APP/PS1 mice led to a reduction of Aβ plaque burden, whereas TREM2 KO had no effect on plaque levels in younger APP/PS1 mice at 4- or 7-months of age [36].

Consistent with prior reports, the 5XFAD × TREM2^−/−^ mice in our study had a significant reduction of microglial clustering around Aβ plaques. 5XFAD × TREM2^+/−^ mice had a modest, non-significant decrease of plaque-associated microglia, with significantly more of these microglia than 5XFAD × TREM2^−/−^ mice. In this regard, a recent study found that TREM2 KO caused a significant reduction of plaque-associated microglia in bigenic mice expressing both tau and APP transgenes, whereas microglia expressing a single copy of TREM2 showed normal plaque interaction [27]. It is thus notable that AD-tau injected 5XFAD × TREM2^+/−^ mice showed an increase of NP tau pathology relative to 5XFAD × TREM2^+/+^ mice, including a trend toward greater NP tau than was observed in 5XFAD × TREM2^−/−^ mice. These data, like those from the PLX3397-treated 5XFAD mice, suggest that the extent of microglia clustering around plaques is not entirely predictive of plaque-associated neuritic damage. DAM gene expression profiling revealed that the 5XFAD × TREM2^+/−^ mice had a reduction of Stage 2 DAM markers that was roughly half the magnitude observed in the 5XFAD × TREM2^−/−^ mice. Moreover, microarray analyses of brain mRNA revealed significant differences between the 5XFAD × TREM2^−/−^ and 5XFAD × TREM2^+/−^ mice, including changes in pathways associated with microglial function and activation. Thus, although the TREM2^+/−^ microglia clearly show differences compared to TREM2^−/−^ microglia in gene expression and degree of plaque engagement, the extent of NP tau formation in the 5XFAD × TREM2^+/−^ mice is equal to or somewhat greater than in 5XFAD × TREM2^−/−^ mice. A somewhat similar trend was previously observed, where TREM2 haploinsufficiency was found to be more detrimental than TREM2 KO in the development of tau pathology in a mouse model with mutant tau over-expression [35]. The TREM2^+/−^ microglia in these tau transgenic mice had increased expression of inflammatory proteins and complement transcripts relative to TREM2^−/−^ microglia. Gene ontology analyses of microarray data from our studies revealed significant differences between 5XFAD × TREM2^−/−^ and 5XFAD × TREM2^+/−^ mice in pathways related to microglial activation in the cortex and hippocampus, although cytokines and complement proteins were not among the most significant differentially expressed mRNA in our study.

The data reported here are generally aligned with another recent study that reported an increase of NP tau pathology after intracerebral AD-tau injection into APP/PS1 × TREM2^−/−^ mice, as well as in APP/PS1 mice expressing human R47H TREM2 in the absence of endogenous mouse TREM2 [28]. APP × TREM2^+/−^ mice were not examined in this study, and in this regard R47H TREM2 microglia may more closely resemble TREM2^−/−^ than TREM2^+/−^ microglia since the R47H TREM2 transcripts undergo atypical mRNA splicing in mice that results in a premature stop codon and reduced TREM2 protein expression [49]. Thus, heterozygous TREM2 mice may serve as a representative model of the single copy loss-of-function TREM2 variants that cause increased risk of AD and may lead to somewhat different results and interpretations than would be obtained with TREM2 KO mice, as disclosed here.

## Supplementary Information

**Additional file 1.** Supplemental figures and tables.

## Data Availability

Study data will be made available upon reasonable request.

## Abbreviations

AD: Alzheimer’s disease
CSFR1: Colony-stimulating factor receptor 1
DAM: Disease-associated microglia
KO: Knockout
MGnD: Microglia with a neurodegenerative phenotype
MT: Microtubule
NP: Neuritic plaque
