# Supplementary material for: Effects of microglial depletion and TREM2 deficiency on Aβ plaque burden and neuritic plaque tau pathology in 5XFAD mice
Source: Acta Neuropathol Commun. 2021 Sep 9;9:150. doi: 10.1186/s40478-021-01251-1 (PMC8428059; doi:10.1186/s40478-021-01251-1)
Supplement: Supplementary file 1 — Additional file 1. Supplemental figures and tables. [file 40478_2021_1251_MOESM1_ESM.pdf]

Supplementary Material

Effects of microglial depletion and TREM2 deficiency on Aβ plaque burden and neuritic plaque tau pathology in 5XFAD mice

Argyro Thalia Delizannis[1], Annelies Nonneman[2], Wangchen Tsering[1], An De Bondt[3], Ilse Van den Wyngaert[3], Bin Zhang[1], Emily Meymand[1], Modupe F. Olufemi[1], Pyry Koivula[1], Shaniya Maimaiti[1], John Q Trojanowski[1], Virginia M-Y Lee[1] and Kurt R. Brunden[1,*]

[1]Center for Neurodegenerative Disease Research, Perelman School of Medicine, University of Pennsylvania, 3600 Spruce St., Philadelphia, PA 19104

[2]Neurosience, Janssen Research & Development, Janssen Pharmaceutica NV (Division of Johnson & Johnson), Turnhoutseweg 30, 2340 Beerse, Belgium

[3]Discovery Sciences, Janssen Research & Development, Janssen Pharmaceutica NV (Division of Johnson & Johnson), Turnhoutseweg 30, 2340 Beerse, Belgium

*Corresponding Author:

kbrunden@upenn.edu
215-615-5262
Fax: 215-349-5909

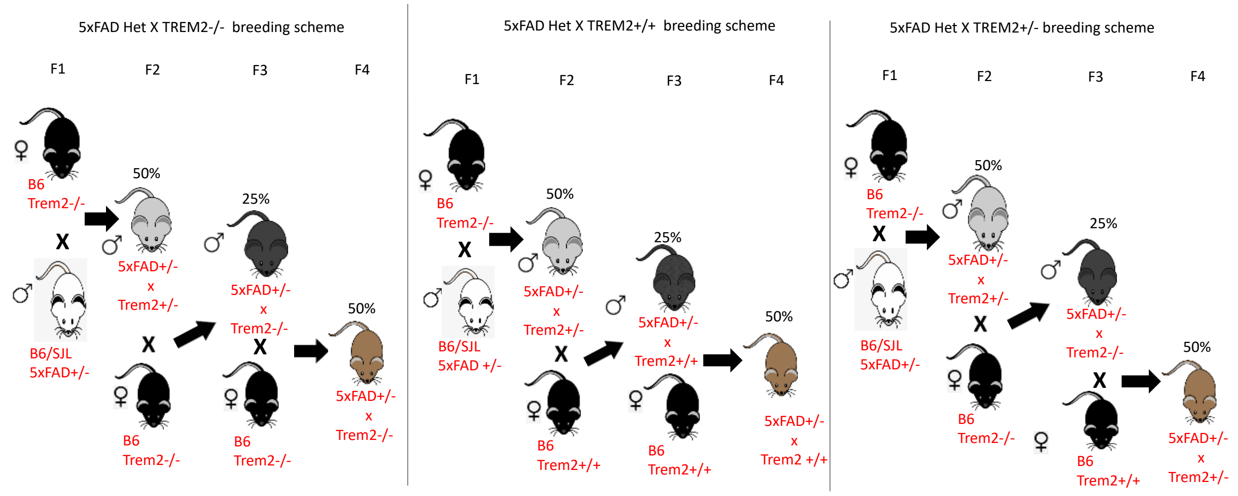

Figure S1. Schematic of the breeding scheme utilized to generate 5XFAD mice with differing TREM2 genotype on identical genetic backgrounds.

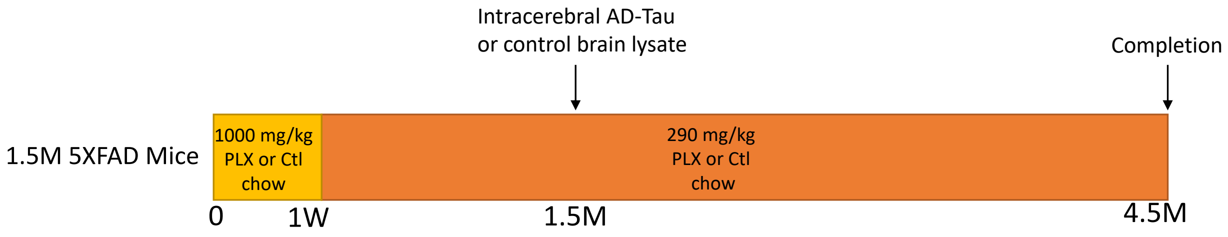

Figure S2. Schematic of study in which chow containing PLX3397 (PLX) or control (Ctl) chow were provided to 1.5-month old 5XFAD mice before and then after intracerebral injection of AD-tau or control brain lysate, for a total of 4.5 months (mice were 6.0-months old at study completion).

A.

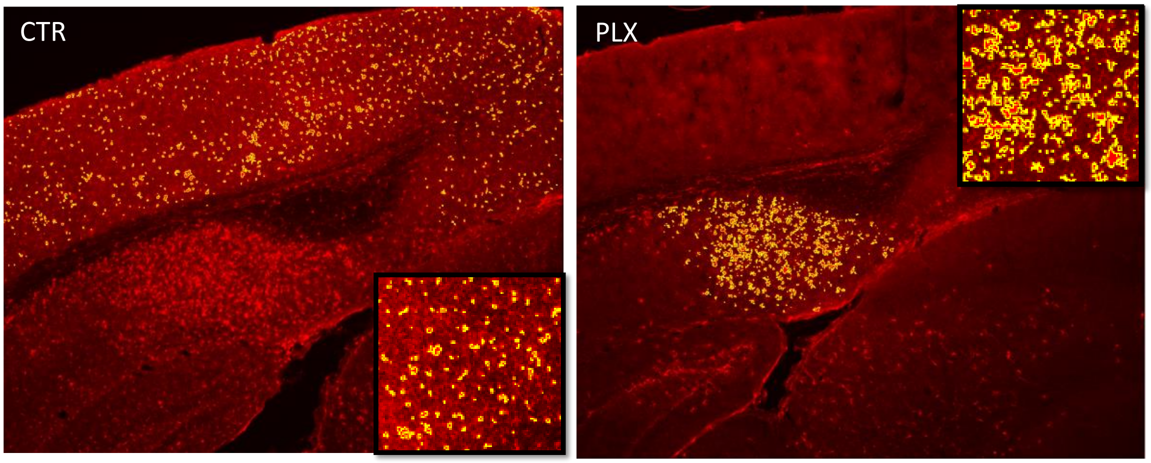

B.

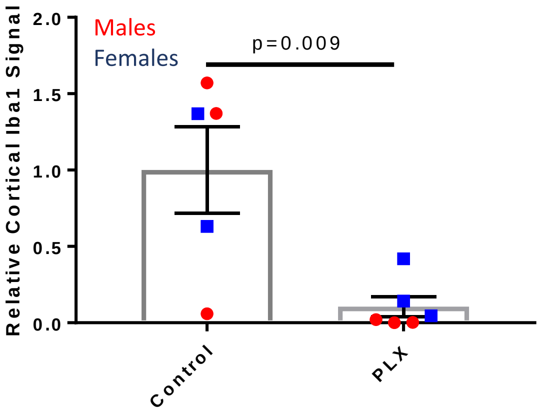

Figure S3. PLX3397 treatment caused significant reduction in brain microglia in both AD-tau- and control extract-injected 5XFAD mice. **A**. Example of Iba1-positive microglial quantification in cortex (left) and subiculum (right) of AD-tau-injected 5XFAD mice. After applying a threshold, positive microglia that are in the focal plane are show in yellow-to-red, representing low-to-high fluorescence signal. All positive Iba1 signal above the threshold was quantified (area x average density). **B**. Iba1-positive microglia were greatly reduced in the cortex of control extract-injected, PLX3397-treated 5XFAD mice. Data represent the sum of values obtained from two different bregma levels, with one female control mouse was excluded since brain sections were not available at both bregma levels.

A.

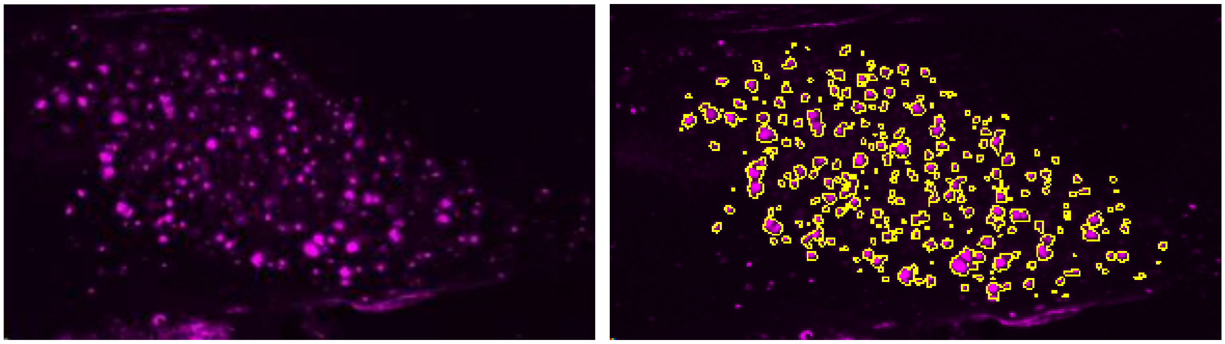

B.

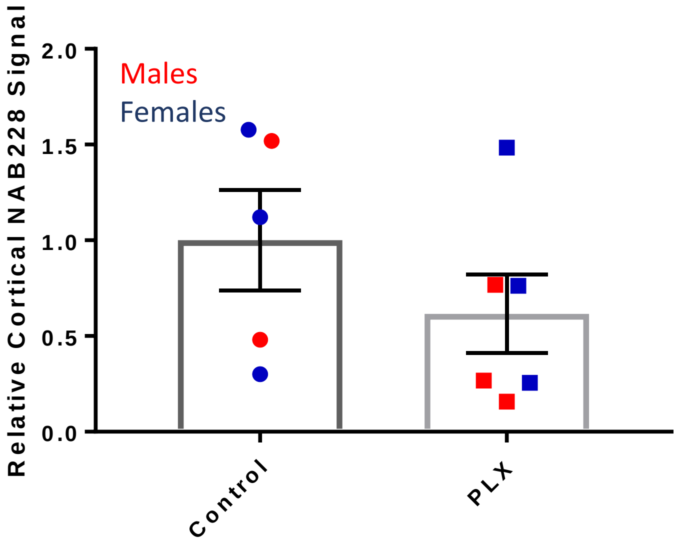

Figure S4. Cortical Aβ plaque IF staining and quantification. **A**. Representative example of the method used to quantify NAB228-positive Aβ plaques in the subiculum, with the IF image shown on the left and the same image shown on the right after applying a threshold. **B**. Quantification of cortical Aβ plaques in 5XFAD mice that had received intracerebral injections with control brain extract. One male control mouse was excluded since matched brain sections were not available.

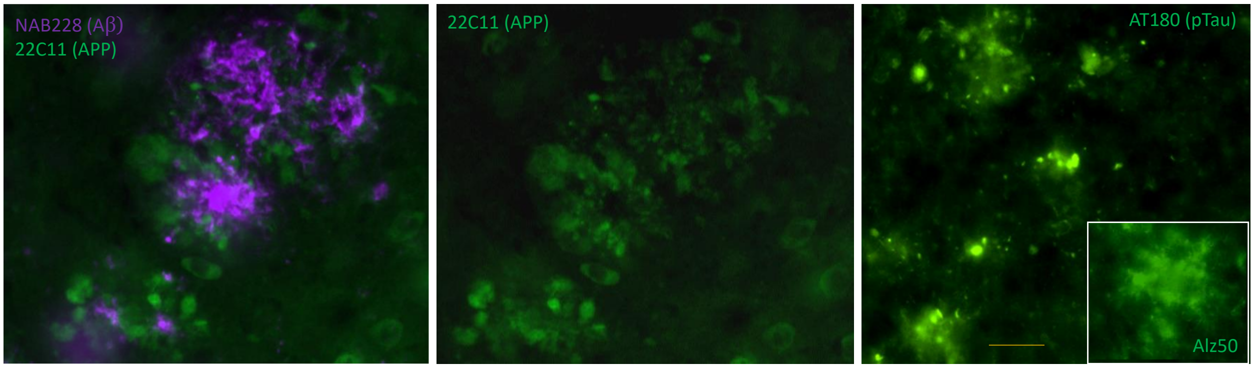

Figure S5. Plaque-associated dystrophic processes in AD-tau-injected 5XFAD x TREM2$^{+/-}$ mice are recognized by AT180 and Alz50 antibodies. Sections from a 5XFAD x TREM2$^{+/-}$ male mouse were dual stained with NAB228 (Aβ) and 22C11 (APP) antibodies (left panel), revealing plaque-associated neuritic dystrophy (22C11 staining only in middle panel). NP tau staining was observed when sections from a 5XFAD x TREM2$^{+/-}$ male mouse were stained with the AT180 (pTau 231) antibody (right panel), as well as with the Alz50 (misfolded tau) antibody (right panel; inset).

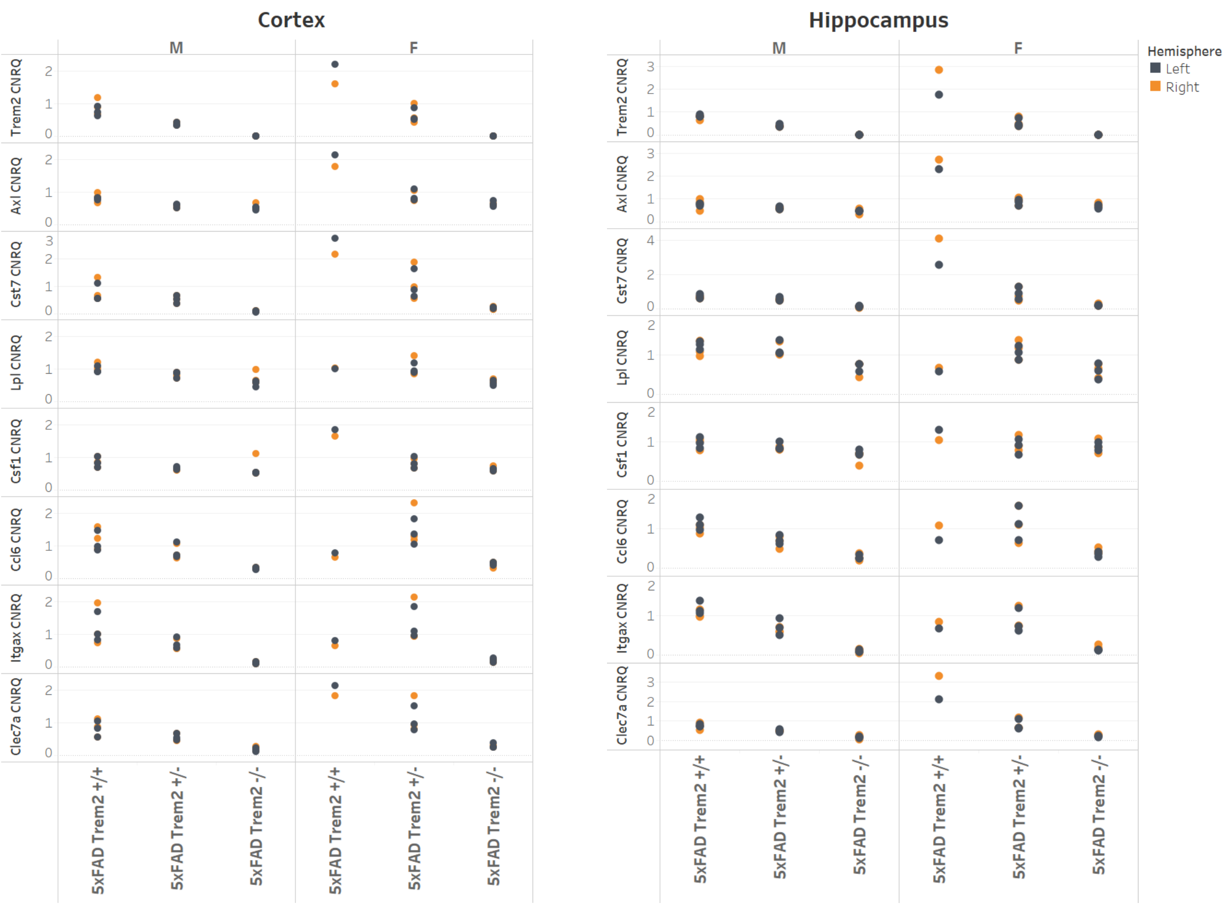

Figure S6. Reduced TREM2 expression led to reduced mRNA expression of DAM stage 2 markers in AD-tau-injected 5XFAD mice, in both males (M) and females (F), and in left (black circles) and right (orange circles) hemisphere samples from cortex and hippocampus.

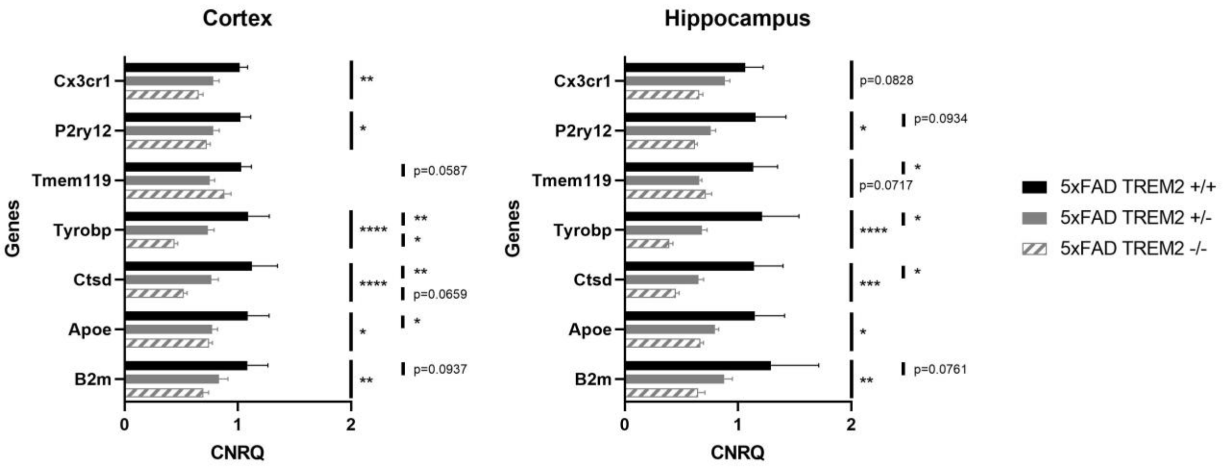

Figure S7. Reduced TREM2 expression led to a modest reduction of the mRNA expression of DAM stage 1 markers in AD-tau-injected 5XFAD mice. qPCR analysis of DAM stage 1 genes in cortex and hippocampus samples of AD-tau-injected 5XFAD x TREM2$^{+/+}$, 5XFAD x TREM2$^{+/-}$ and 5XFAD x TREM2$^{-/-}$ mice. Male (n=3) and female (n=3, except n=1 for 5XFAD x TREM2$^{+/+}$) mice were pooled, and samples of left and right hemisphere were used as individual data points (data by sex and hemisphere can be found in Fig. S8). *p<0.05, **p<0.01, ***p<0.001, ****p<0.0001.

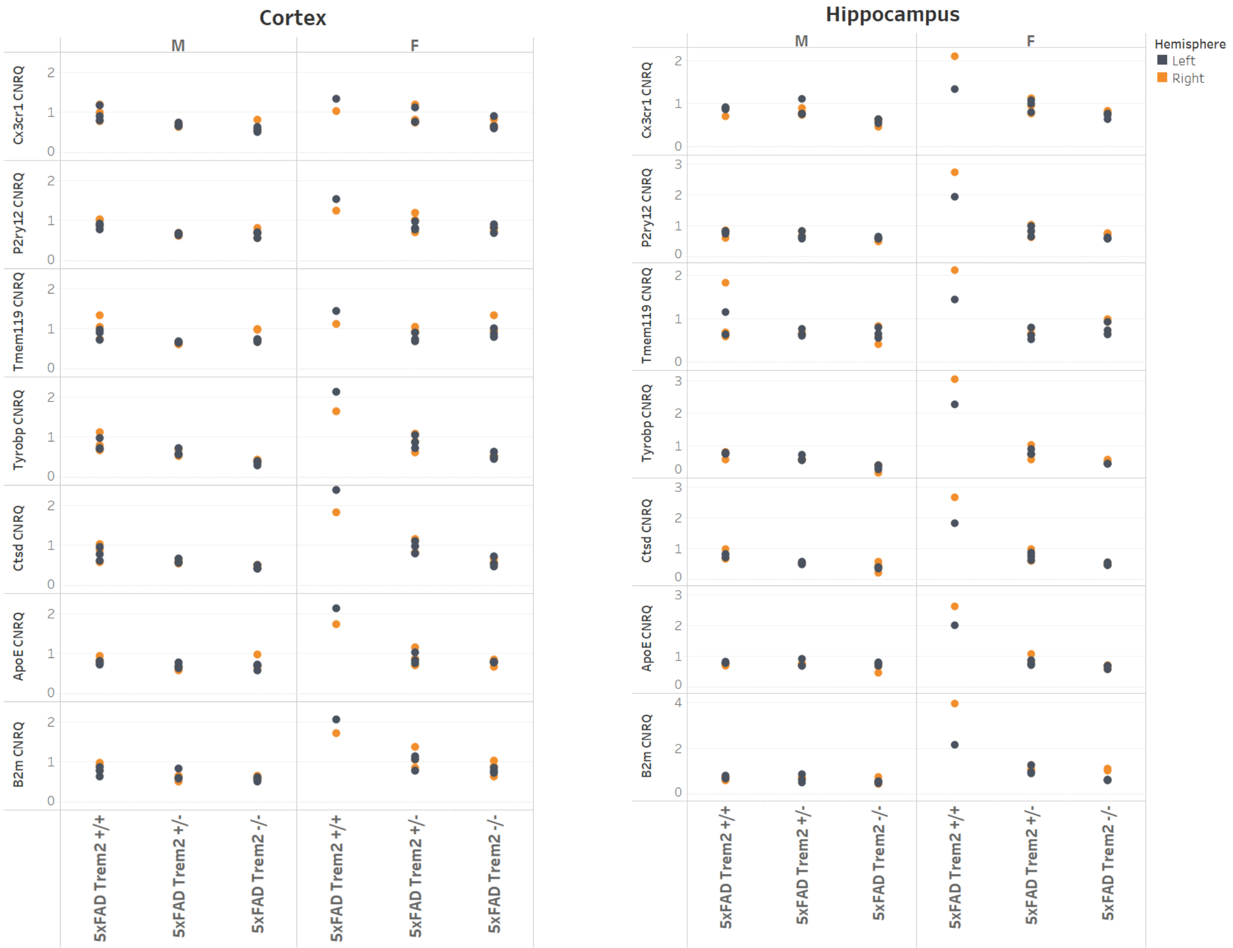

Figure S8. Reduced TREM2 expression led to a modest reduction of the mRNA expression of DAM stage 1 markers in AD-tau-injected 5XFAD mice, in both males (M) and females (F), and in left (black circles) and right (orange circles) hemisphere samples from cortex and hippocampus.

Cortex Comparison	nUp	nDown
TREM2$^{+/-}$ vs TREM2$^{+/+}$	367	344
TREM2$^{-/-}$ vs TREM2$^{+/+}$	593	402
TREM2$^{-/-}$ vs TREM2$^{+/-}$	136	36
**Hippocampus Comparison**	**nUp**	**nDown**
TREM2$^{+/-}$ vs TREM2$^{+/+}$	2021	1201
TREM2$^{-/-}$ vs TREM2$^{+/+}$	2636	1458
TREM2$^{-/-}$ vs TREM2$^{+/-}$	17	5

Table S1. Summary of gene expression changes in the cortex and hippocampus when comparing AD-tau-injected 5XFAD mice with different TREM2 genotypes. Listed values pertain to genes with multiple testing adjusted p-value <0.05.

Cortex

rank	id	size	pvalue	description
1	GO:0010257	42	5.00E-12	NADH dehydrogenase complex assembly
2	GO:0032981	42	5.00E-12	mitochondrial respiratory chain complex I assembly
3	GO:0006119	80	2.10E-10	oxidative phosphorylation
4	GO:0022904	67	3.80E-10	respiratory electron transport chain
5	GO:0042773	48	4.30E-10	ATP synthesis coupled electron transport
6	GO:0022900	71	4.40E-10	electron transport chain
7	GO:0042775	47	4.60E-10	mitochondrial ATP synthesis coupled electron trans
8	GO:0033108	70	1.30E-09	mitochondrial respiratory chain complex assembly
9	GO:1903978	19	1.40E-07	regulation of microglial cell activation
10	GO:1903979	7	5.70E-07	negative regulation of microglial cell activation

Hippocampus

rank	id	size	pvalue	description
1	GO:0034241	5	5.80E-08	positive regulation of macrophage fusion
2	GO:0034239	6	1.70E-07	regulation of macrophage fusion
3	GO:1903978	19	2.80E-07	regulation of microglial cell activation
4	GO:1900223	5	7.70E-07	positive regulation of amyloid-beta clearance
5	GO:0061890	5	9.60E-07	positive regulation of astrocyte activation

Table S2. Most affected gene ontology biological processes ($p < 1 \times 10^{-6}$) in the cortex and hippocampus between the AD-tau-injected 5XFAD x TREM2$^{+/-}$ and 5XFAD x TREM$^{-/-}$ mice.

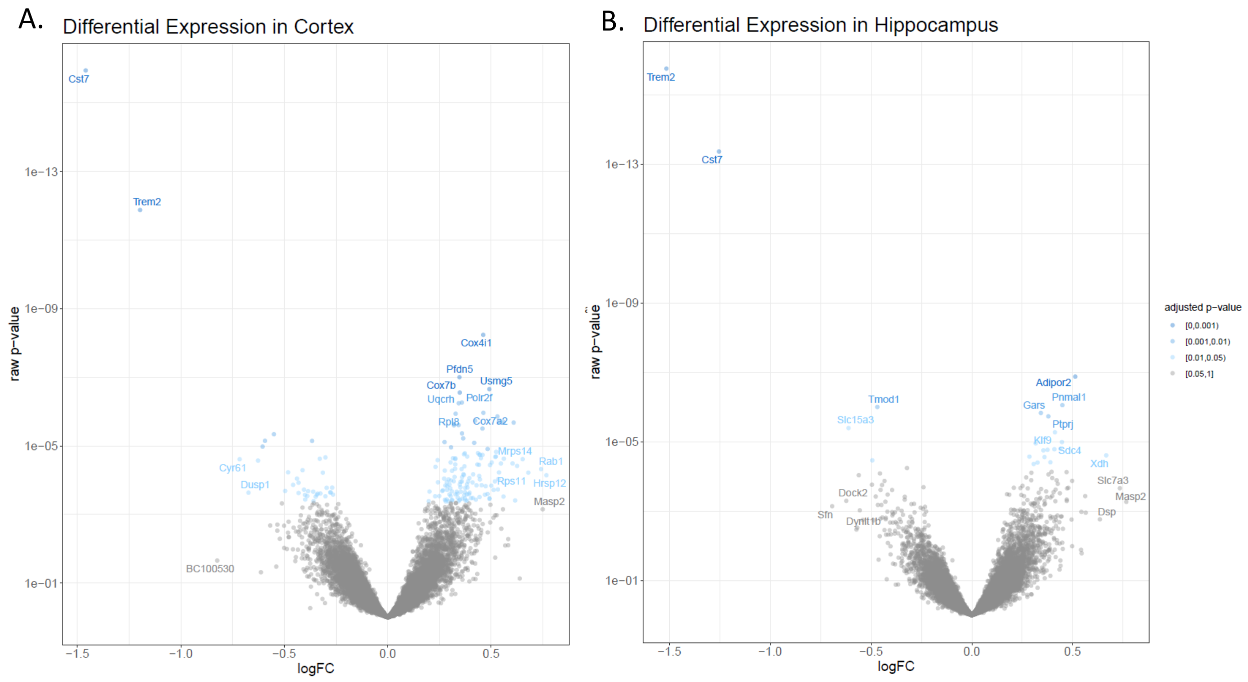

Figure S9. Differential expression differences between the AD-tau-injected 5XFAD x TREM2$^{-/-}$ and 5XFAD x TREM2$^{+/-}$ mice in the cortex (**A**) and hippocampus (**B**). Genes indicated in blue are significant after correction for multiple testing, with the most outlying genes annotated with their gene symbol.

A.

SYMBOL	logFC	adj.P.Val	GENEFAMILY	GENENAME
Cst7	-1.5	2.30E-12	Secreted	cystatin F (leukocystatin)
Trem2	-1.2	1.30E-08		triggering receptor expressed on myeloid c
Cox4i1	0.5	3.90E-05	transporter	cytochrome c oxidase subunit IV isoform 1
Pfdn5	0.3	5.00E-04		prefoldin 5
Usmg5	0.5	8.90E-04		upregulated during skeletal muscle growth
Cox7b	0.3	9.40E-04	transporter	cytochrome c oxidase subunit VIIb
Polr2f	0.4	1.40E-03		polymerase (RNA) II (DNA directed) polype
Uqcrh	0.3	1.40E-03	transporter	ubiquinol-cytochrome c reductase hinge pr
Cox7a2	0.5	2.30E-03	transporter	cytochrome c oxidase subunit VIIa 2
Rpl8	0.3	2.30E-03		ribosomal protein L8
2010107E04Rik	0.5	2.50E-03		RIKEN cDNA 2010107E04 gene
Pfdn2	0.4	2.80E-03		prefoldin 2
Mettl9	0.5	2.80E-03	Secreted	methyltransferase like 9
Snrpd3	0.6	2.80E-03		small nuclear ribonucleoprotein D3
Psmb4	0.6	2.80E-03	peptidase	proteasome (prosome, macropain) subunit
Ube2k	0.3	2.80E-03		ubiquitin-conjugating enzyme E2K
Mdh1	0.3	2.80E-03		malate dehydrogenase 1, NAD (soluble)
Psma1	0.3	2.80E-03	peptidase	proteasome (prosome, macropain) subunit
Atp5b	0.5	3.30E-03	transporter	ATP synthase, H+ transporting mitochondri
Ndufa13	0.4	4.40E-03		NADH dehydrogenase (ubiquinone) 1 alpha

B.

SYMBOL	logFC	adj.P.Val	GENEFAMILY	GENENAME
Trem2	-1.5	3.50E-12		triggering receptor expressed on myeloid cel
Cst7	-1.3	4.30E-10	Secreted	cystatin F (leukocystatin)
Adipor2	0.5	8.90E-04		adiponectin receptor 2
Pnmal1	0.4	4.00E-03		PNMA-like 1
Tmod1	-0.5	4.00E-03		tropomodulin 1
Gars	0.3	5.00E-03		glycyl-tRNA synthetase
Ptprj	0.4	5.30E-03	phosphatase	protein tyrosine phosphatase, receptor type
Slc15a3	-0.6	1.00E-02	transporter	solute carrier family 15, member 3
Klf9	0.4	1.20E-02		Kruppel-like factor 9
Sdc4	0.4	2.00E-02		syndecan 4
Ndfip1	0.3	2.00E-02		Nedd4 family interacting protein 1
Mertk	0.4	2.40E-02	kinase	c-mer proto-oncogene tyrosine kinase
Ndufb8	0.4	2.40E-02		NADH dehydrogenase (ubiquinone) 1 beta su
Atp1b2	0.4	2.40E-02	Secreted	ATPase, Na+/K+ transporting, beta 2 polypep
Aimp1	0.4	2.40E-02		aminoacyl tRNA synthetase complex-interac
Xdh	0.7	3.10E-02		xanthine dehydrogenase
Mt2	0.3	3.10E-02		metallothionein 2
Dnajc3	0.4	3.10E-02	Secreted	DnaJ (Hsp40) homolog, subfamily C, membe
Col6a1	-0.5	3.70E-02	Secreted	collagen, type VI, alpha 1
Lrrn1	0.4	3.80E-02		leucine rich repeat protein 1, neuronal

Table S3. Genes with the most significant differential expression between AD-tau-injected 5XFAD x TREM2$^{-/-}$ and 5XFAD x TREM2$^{+/-}$ mice in the cortex (**A**) and hippocampus (**B**).
